# Meflin confers antifibrotic properties to intestinal fibroblasts in inflammatory bowel disease

**DOI:** 10.1172/JCI192804

**Published:** 2026-05-19

**Authors:** Jingxi Mu, Keiko Maeda, Tadashi Iida, Shinji Mii, Nobutoshi Esaki, Yukihiro Shiraki, Yasuyuki Mizutani, Masanao Nakamura, Takeshi Yamamura, Tsunaki Sawada, Eri Ishikawa, Kentaro Murate, Takashi Hirose, Kazuhiro Furukawa, Akina Oishi, Haruhiko Suzuki, Takayoshi Kishida, Goro Nakayama, Mitsuhiro Fujishiro, Hiroki Kawashima, Atsushi Enomoto

**Affiliations:** 1 Department of Gastroenterology and Hepatology,; 2 Department of Pathology,; 3 Department of Endoscopy,; 4 Department of Immunology, and; 5 Department of Gastroenterological Surgery, Nagoya University Graduate School of Medicine, Nagoya, Japan.; 6 Department of Gastroenterology, Graduate School of Medicine, The University of Tokyo, Tokyo, Japan.; 7 Center for One Medicine Innovative Translational Research, Gifu University Institute for Advanced Study, Gifu, Japan.

**Keywords:** Gastroenterology, Inflammation, Fibrosis, Inflammatory bowel disease, Therapeutics

## Abstract

Dysfunctional intestinal fibrosis is an irreversible complication of Crohn’s disease (CD). The complex heterogeneity of intestinal mesenchymal cells makes it difficult to understand the pathogenesis of intestinal fibrosis. Previously, we identified Meflin as a marker of fibroblast subsets. This study aimed to explore the role of Meflin-positive fibroblasts in intestinal fibrogenesis and investigate the potential of pharmacological control of Meflin expression as a treatment for patients with CD. Our results indicated that Meflin expression was upregulated in fibroblasts at the early stage of fibrosis but was downregulated in established fibrosis in both patients with CD and 2 different mouse models, which are the chronic dextran sodium sulfate (DSS) model and an IL-10–deficient model that spontaneously develops intestinal inflammation. Meflin-deficient mice exacerbated intestinal fibrosis with dysregulated expression of noncanonical Wnt ligand WNT5A and its receptor ROR2. Pharmacologically induced Meflin expression through the administration of a synthetic retinoid reversed intestinal fibrosis in the DSS model and suppressed profibrotic protein secretion in fibroblasts isolated from patients with CD. Our findings indicate that Meflin-positive fibroblasts represent a functional subpopulation that suppresses intestinal fibrosis. Augmentation of Meflin expression shows antifibrotic effects and holds promise as a therapeutic approach for intestinal fibrosis in patients with CD.

## Abstract 2

Pharmacologically inducing Meflin expression significantly reversed intestinal fibrosis in experimental models, highlighting Meflin as a promising therapeutic target for treating fibrosis in Crohn’s disease patients.

## Graphical abstract

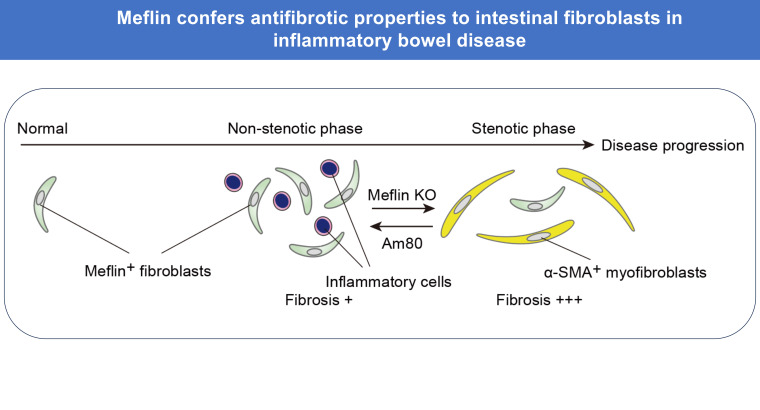

## Introduction

Dysfunctional structural intestinal fibrosis, characterized by intestinal strictures and organ failure, is a serious complication in patients with Crohn’s Disease (CD) (1, 2). Despite recent advances in antiinflammatory therapies targeting multiple pathways, biologics have not reduced surgery rates for intestinal fibrosis in these patients (3). The lack of specific antifibrotic treatments for patients with CD has led to the traditional view that intestinal fibrosis is an irreversible process that ultimately requires surgical intervention, owing to severe fibrosis-related complications (3). Therefore, it is necessary to develop new drugs that specifically target fibrogenesis.

Colonic mesenchymal heterogeneity is crucial for maintaining homeostasis and facilitating tissue repair and plays an essential role at different stages of fibrogenesis in patients with CD through different subpopulations (4, 5). Intestinal fibroblasts are major signal transducers in the strictured bowel of patients with CD (6). Recent single-cell RNA sequencing (scRNA-seq) approaches have revealed several heterogeneous fibroblast populations that are differentially involved in intestinal fibrosis (7). For example, the scRNA atlas of tissues obtained from patients with CD with full-thickness strictures showed that fibroblasts in areas with and without strictures differ, with a notable increase in the expression of specific matrix metalloproteases and the noncanonical Wnt regulator *WNT5A* (8).

However, the specific fibroblast subsets involved in the development and suppression of fibrosis remain unknown. Therefore, it is essential to identify the functional subpopulations of intestinal fibroblasts involved in the progression of fibrogenesis and develop targeted strategies.

Our previous research identified Meflin, a glycosylphosphatidylinositol-anchored membrane and secreted protein encoded by the immunoglobulin superfamily containing the leucine-rich repeat (*ISLR*) gene, as a marker of mesenchymal stromal cells and fibroblasts that reside in various tissues and are critical for maintaining their undifferentiated state (9). Meflin lacks intrinsic enzymatic activities to directly regulate intracellular signaling, suggesting that its biological functions are primarily mediated through interacting with other proteins in the extracellular space or acting on neighbor cells. Indeed, stromal-derived Meflin has been reported to promote intestinal regeneration by suppressing Hippo signaling in epithelial cells (10), supporting its paracrine mode of action. In skeletal muscle regeneration, Meflin has been shown to associate with the Wnt signaling effector Dishevelled-2 to modulate canonical Wnt signaling (11). Thus, Meflin engages with distinct signaling partners in a context-dependent manner and functions as an extracellular regulator that integrates stromal signaling.

Previous studies, including our own, showed that Meflin plays an important role in promoting muscle and intestinal regeneration, cardiac tissue repair, and suppression of cancer progression by regulating fibroblast heterogeneity (10–15). Meflin suppresses tissue fibrosis by suppressing the transition of fibroblasts to the myofibroblastic phenotype through the augmentation of the activity of bone morphogenetic protein 7 (BMP7), which is known to counteract TGF-β activity, which is essential for fibrosis, and suppression of the collagen cross-linking enzyme lysyl oxidase (13, 14, 16). We recently identified Am80 (tamibarotene), a synthetic retinoid, which effectively induces Meflin expression and suppresses stromal fibrosis in cancer mouse models (14). However, it remains unclear how Meflin-positive fibroblasts are involved in the development of intestinal fibrosis, and the potential therapeutic effect via regulating Meflin expression in inflammatory bowel diseases (IBD), including CD.

This study aimed to explore the potential role of Meflin in intestinal fibrogenesis and develop a treatment strategy for intestinal fibrosis by pharmacologically inducing Meflin expression in patients with CD.

## Results

### Meflin expression in intestinal stromal cells of fibrotic tissues in patients with CD.

**Figure 1:**
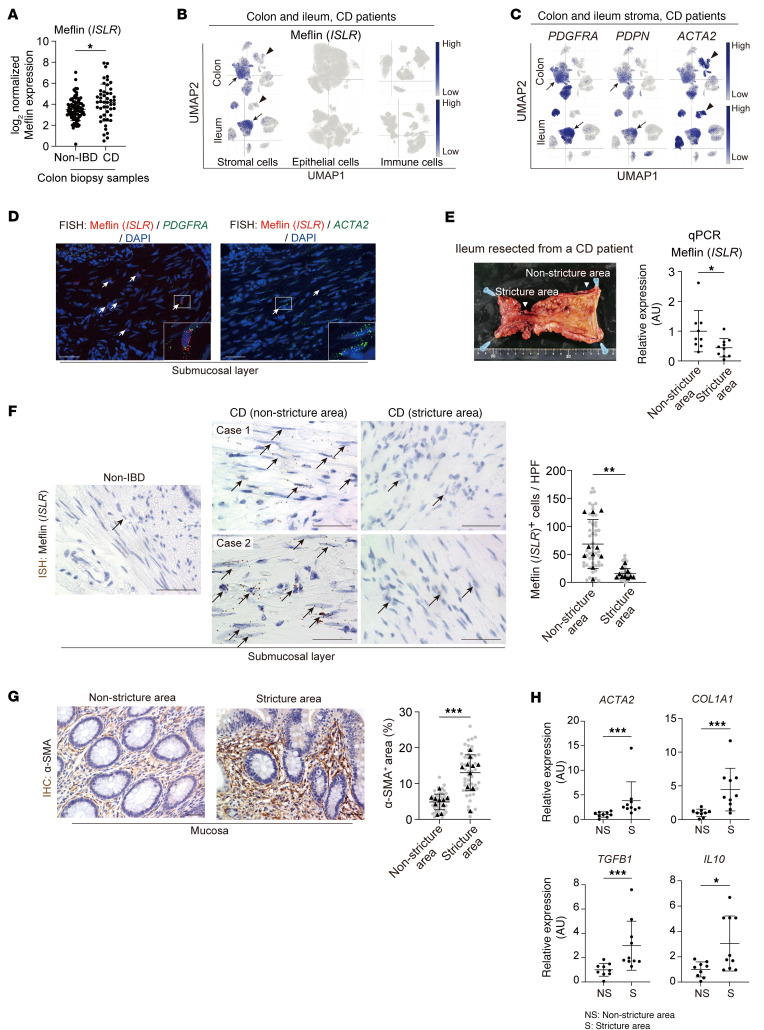
Downregulation of Meflin expression in fibroblasts in the intestinal stricture regions. (**A**) Normalized Meflin (*ISLR*) mRNA expression in colon tissue biopsies obtained from non-IBD healthy individuals (*n* = 80) and patients with CD (*n* = 48). (**B**) UMAP feature plots show Meflin (*ISLR*) expression in stromal cell clusters but not in epithelial and immune cells. (**C**) UMAP feature plots showing normalized *PDGFRA*, *PDPN,* and *ACTA2,* and expressions in the stromal cell cluster. The arrows indicate cell clusters that coexpress Meflin, *PDGFRA*, and *PDPN*. The solid arrowhead indicates the cluster in the top right, which shows high *ACTA2* expression but lacks Meflin expression. (**D**) FISH analysis for Meflin (red) and either *PDGFRA* (green, left panel) or *ACTA2* (green, right panel) in the submucosal layer of the CD-affected intestine. Boxed areas are magnified in insets. Arrows denote double-positive cells. (**E**) Representative macroscopic image of the ileum surgically resected from a patient with CD. Stricture and nonstricture areas are indicated with arrowheads (left panel) and quantitative real-time polymerase chain reaction (qPCR) analysis for Meflin (right panel, *n* = 10 pairs). (**F**) Representative images of Meflin ISH in intestinal tissue sections from non-IBD and nonstricture and stricture areas of patients with CD (Case 1 and 2) and their quantification. Arrows denote Meflin^+^ cells. (**G**) Intestinal tissue sections of patients with CD were analyzed by IHC for α-SMA, followed by quantification of α-SMA-positive areas. (**H**) qPCR analysis of the intestines from patients with CD (*n* = 10 pairs). (**F** and **G**) 5 high-power fields (HPFs) per area were quantified for each patient. Small gray dots indicate individual HPFs, and black triangles indicate patient-level means used for statistical analysis. Paired samples from 10 patients were analyzed. Scale bars: 40 μm. 2-tailed *t* tests. *P < 0.05; **P < 0.01; ***P < 0.001.

We analyzed NCBI GEO, a publicly available database (GSE165512, GSE83687) (17, 18) using DESeq2 and found significant upregulation of Meflin in tissue biopsy samples taken from the inflamed area of the colon of patients with CD compared with people who were in the non-IBD control group (Figure 1A). Analysis of a publicly available scRNA-seq dataset available from the Broad Institute Single Cell Portal (SCP1884) obtained from cells isolated from both the ileum and colon of patients with CD (19), revealed that Meflin expression was limited to the intestinal stromal cells, with no expression in epithelial or immune cell populations (Figure 1B and Supplemental Figure 1; supplemental material available online with this article; https://doi.org/10.1172/JCI192804DS1). Uniform manifold approximation and projection (UMAP) clustering analysis revealed that Meflin expression was enriched in stromal cells positive for *PDGFRA* and *PDPN*, canonical fibroblast marker genes encoding platelet-derived growth factor receptor α (PDGFRα) and podoplanin, respectively. In contrast, the expression of *ACTA2*, which encodes the myofibroblast marker α-SMA, was enriched in different stromal clusters that may also comprise pericytes and smooth muscle cells (Figure 1, B and C). Subsequent fluorescence in situ hybridization (FISH) analysis confirmed that most Meflin^+^ stromal cells coexpressed *PDGFRA* but showed limited *ACTA2* expression in the submucosal layer of CD-affected intestine (Figure 1D). Based on these findings and given the central role of intestinal stromal cells in fibrosis, we hypothesized that Meflin may play a crucial role in fibrosis in patients with CD.

Analysis of a publicly available transcriptomic dataset from NCBI Gene Expression Omnibus (GEO; GSE158952) of intestinal biopsy samples from patients with CD (20) showed that Meflin expression was lower in the ileum, which is more prone to stricture formation, than the rectum (Supplemental Figure 2A). Real-time quantitative polymerase chain reaction (RT-qPCR) analysis of tissues from surgically resected intestines of patients with CD showed that Meflin expression was significantly higher in nonstricture areas than in stricture areas with fibrosis (Figure 1E). Meflin expression was increased in the nonstricture areas of patients with CD compared with tissues from people in the non-IBD control group both at the mRNA and protein levels, whereas it was significantly downregulated in the stricture areas that were strongly positive for α-SMA (Figure 1, F and G and Supplemental Figure 2, B and C). This was further corroborated by analysis of the previously published RNA-seq dataset from Mukherjee et al. (8), which showed that Meflin expression was downregulated in the inflamed and stricture areas (Supplemental Figure 2D). Differences in the expression of fibroinflammatory markers such as *ACTA2*, *COL1A1*, *TGFB1*, and *IL-10* between nonstricture and stricture areas were also confirmed by RT-qPCR (Figure 1H).

To investigate the mechanism underlying the regulation of Meflin expression, we treated human intestinal fibroblasts isolated from patients with CD with TGF-β, which resulted in an upregulation of *ACTA2* and significant downregulation Meflin expression (Supplemental Figure 2, E and F). The Pearson correlation analysis of the gene expression profiles of balloon-assisted enteroscopic tissue biopsy samples taken from nonstricture ileal areas of patients with CD (*n* = 24, Supplemental Table 1) showed significant correlations between Meflin and *TGFB1* and *COL1A1* (Supplemental Figure 2G). Taken together, these findings suggest the involvement of Meflin downregulation in fibroblasts in the progression of intestinal fibrosis in patients with CD.

### Meflin deficiency aggravates intestinal fibrosis in the DSS-induced chronic colitis model.

**Figure 2:**
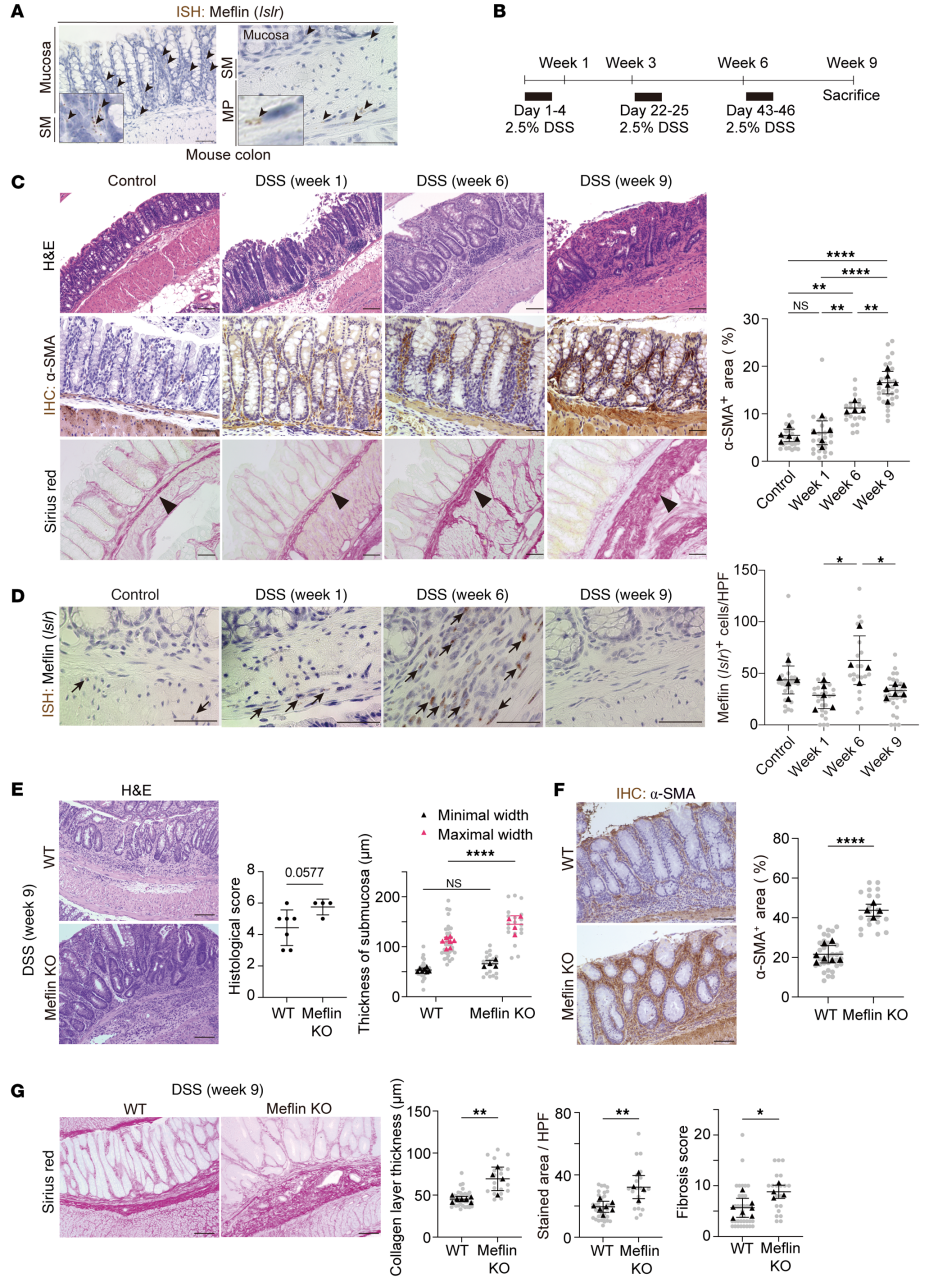
Meflin deficiency aggravates intestinal fibrosis in the DSS-induced colitis model. (**A**) ISH for Meflin (*Islr*) in the mouse colon. Arrowheads denote Meflin^+^ cells. SM, submucosa; MP, musclularis propria. (**B**) Schematic diagram of DSS-induced chronic colitis and fibrosis model. (**C** and **D**) Colonic tissue sections from WT mice at different time points after DSS administration are examined by H&E staining, IHC for α-SMA, Sirius Red staining (**C**), and ISH for Meflin (**D**). Note that muscularis mucosa thickness is increased over time (arrowheads). Quantification (*n* = 3–5/group). Arrows denote Meflin^+^ cells. 1-way ANOVA. (**E**) Representative images of H&E-stained colon sections from WT and Meflin-KO mice after 9 weeks of DSS administration (*n* = 7 and 4 for WT and Meflin-KO mice, respectively), followed by the quantification of submucosa thickness. (**F** and **G**) Representative images of IHC for α-SMA (**F**) and Sirius Red staining (**G**) on colon sections from WT and Meflin-KO mice after DSS administration, followed by the quantifications of α-SMA–positive areas, collagen layer thickness, Sirius Red^+^ areas, and fibrosis score. (**C**–**G**) Five HPFs per area were quantified for each mouse. Small gray dots indicate individual HPFs, and black triangles indicate mouse-level means used for statistical analysis. Scale bars: 40 μm. 2-tailed Student’s *t* tests unless otherwise indicated. *P < 0.05; **P < 0.01; ****P < 0.0001.

Similar to the higher expression in biopsy samples taken from the rectum than in the ileum in patients with CD in a publicly available dataset from NCBI Gene Expression Omnibus (GEO; GSE158952) (20), Meflin expression was more abundant in the mouse colon than in the small intestine (Supplemental Figure 3A). In the colon, Meflin^+^ fibroblasts were located in the subepithelial region and around the crypts within the lamina propria, as well as in the submucosal layer and muscularis propria (Figure 2A).

We first compared the littermates of WT and Meflin-knockout (KO) mice of the same sex and age, which we previously generated (9, 12–14) and found that there were no preexisting differences at baseline in either overall morphology or fibrotic indicators, including the expression of profibrotic and inflammatory genes such as *Tgfb1, Acta2, Col3a1*, and *Mmp9* (Supplemental Figure 3, B and C). To clarify the involvement of Meflin in inflammation-induced intestinal fibrosis, we used the chronic dextran sodium sulfate–induced (DSS-induced) colitis model, which is widely recognized and used in modeling fibrogenesis of IBD (21–23) (Figure 2B). Progressive intestinal inflammation and fibrosis were confirmed by histological evaluation, immunohistochemistry (IHC) for α-SMA, and Sirius Red staining (Figure 2C). The in situ hybridization (ISH) assay revealed that Meflin expression was initially upregulated, peaking at the early stage of fibrosis approximately 6 weeks after starting DSS administration, but was significantly downregulated once intestinal fibrosis was established at week 9, as evidenced by extensive collagen deposition and α-SMA expression (Figure 2, C and D).

To investigate the potential involvement of Meflin in fibrosis progression, we subjected Meflin-KO mice to the DSS-induced colitis model. Histological analysis at 9 weeks after starting DSS administration revealed that intestinal inflammation in Meflin-KO mice was more prominent than that in WT mice, as assessed by the inflammatory histological scoring system (24) and quantification of the minimum and maximum submucosal thickness (Figure 2E). Correspondingly, intestinal fibrosis was significantly more severe in Meflin-KO mice than in WT mice (Figure 2, F and G). These results suggested that Meflin plays a protective role against the development of inflammation and fibrosis.

### Meflin deficiency accelerates intestinal fibrosis in a spontaneous colitis model induced by IL-10 deficiency.

**Figure 3:**
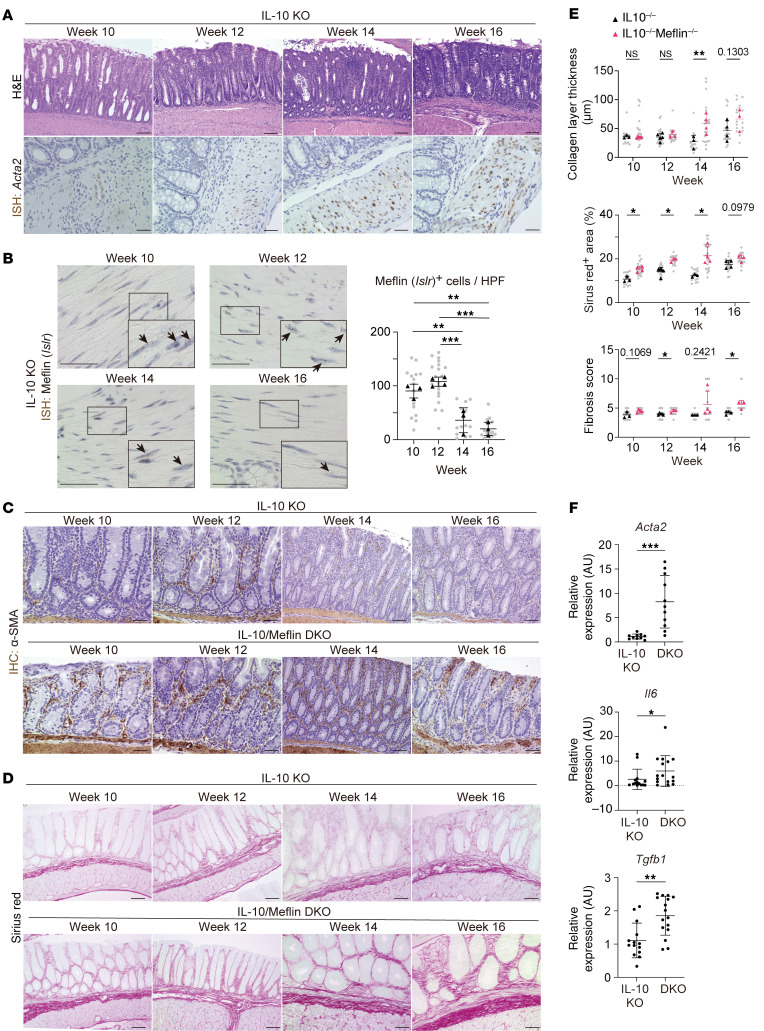
Meflin deficiency accelerates intestinal fibrosis in the IL-10–KO spontaneous colitis model. (**A** and **B**) Colon tissue sections from IL-10–KO mice at weeks 10, 12, 14, and 16 are subjected to H&E staining (upper panels), ISH for *Acta2* (lower panels) (**A**), and ISH for Meflin (**B**), followed by quantification (*n* = 3–5/group). Arrows indicate Meflin-positive cells. (**C**–**E**) Colon tissue sections from IL-10–KO and IL-10/Meflin–DKO mice at weeks 10, 12, 14, and 16 are subjected to IHC for α-SMA (**C**) and Sirius Red staining (**D**), followed by collagen layer thickness, quantification of Sirius Red^+^ areas, and fibrosis score (**E**) (*n* = 3–5/ group). (**F**) qPCR analysis for A*cta2*, I*l6*, and T*gfb1* mRNA in total RNAs isolated from all the colons of IL-10–KO and IL-10/Meflin–DKO mice (*n* = 14 and 16, respectively). Each dot represents an individual sample. 2-tailed Student’s *t* tests. (**B** and **E**). 5 HPFs per area were quantified for each mouse. Small gray dots indicate individual HPFs, and black triangles indicate mouse-level means used for statistical analysis. Scale bars: 40 μm. 1-way ANOVA for **B** and 2-tailed Student’s *t* tests for **E**. *P < 0.05; ** *P* < 0.01; *** *P* < 0.001; ****P < 0.0001.

We then used IL-10–KO mice deficient in the expression of the antiinflammatory cytokine IL-10, which is known to spontaneously develop small intestinal and colonic intestinal colitis and fibrosis without DSS or any other chemical administration (Supplemental Figure 3D) (25, 26), and confirmed the progressive development of intestinal inflammation and fibrosis over time (Figure 3A). ISH analysis of Meflin expression at different time points showed that there were a large number of Meflin^+^ fibroblasts at the early stages of fibrosis development from 10–12 weeks; however, their numbers significantly decreased at later stages from 14–16 weeks (Figure 3B).

We generated IL-10/Meflin double–knockout (DKO) mice (Supplemental Figure 3, D and E) and compared the extent of fibrosis between IL-10–KO and IL-10/Meflin–DKO mice at different time points. A more accelerated fibrogenesis was observed in IL-10/Meflin-DKO mice compared with IL-10–KO mice (Figure 3, C–E, and Supplemental Figure 3F). Consistent with this finding, the expressions of profibrotic and inflammatory genes, such as *Acta2*, *Il6*, and *Tgfb1,* were more dominant in the fibrotic intestine of IL-10/Meflin DKO mice than in IL-10–KO mice (Figure 3F). Our results demonstrate that Meflin deficiency in fibroblasts aggravates the intestinal fibroinflammatory reaction induced by IL-10 deficiency in mice.

### Antifibrotic effect of pharmacological induction of Meflin expression in fibroblasts on intestinal fibrosis.

**Figure 4:**
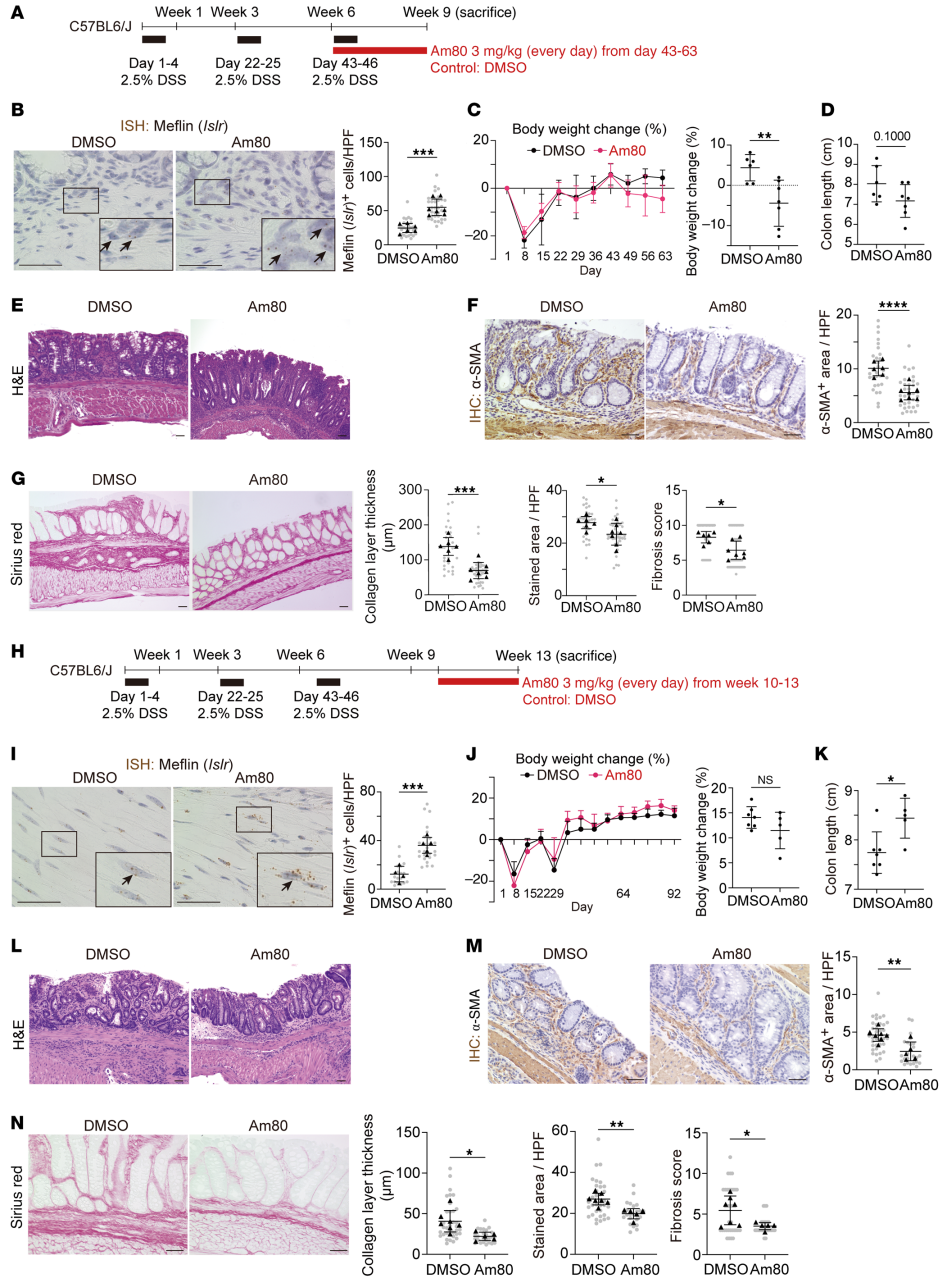
Antifibrotic effect of Am80 administration on intestinal fibrosis in the DSS mouse model. (**A**) Schematic diagram showing the experimental setup. Oral administration of Am80 (3 mg/kg/day) or DMSO every day was initiated simultaneously with the third dose of DSS administration from days 43–63. (**B**) Colon tissue sections from WT mice after Am80 or DMSO administration for 9 weeks are analyzed for Meflin expression using ISH. Boxed areas are magnified in insets. Arrows denote Meflin^+^ cells. (**C** and **D**) Body weight changes over time and colon length at Day 63 are measured. (**E**–**G**) Colon tissue sections after Am80 administration are stained with H&E (**E**), IHC for α-SMA (**F**), and Sirius Red (**G**), followed by collagen layer thickness, quantification of Sirius Red^+^ areas, and fibrosis score. (**F** and **G**) (*n* = 6 and 7 for the control and Am80 groups, respectively). (**H**) Oral administration of Am80 (3 mg/kg/day) or DMSO every day to WT mice from weeks 10 to 13 after the 3 cycles of DSS administration. (**I**) Colon tissue sections are analyzed for Meflin expression using ISH. Boxed areas are magnified in insets. Arrows denote Meflin^+^ cells. (*n* = 4 and 5 for the control and Am80 groups, respectively). (**J** and **K**) Body weight changes over time and colon length at Day 92 are evaluated. (**L**–**N**) Representative images for H&E, IHC for α-SMA, and Sirius Red staining performed in colon tissue sections from WT mice after DSS and subsequent Am80 administrations (*n* = 7 and 5 for the control and Am80 groups, respectively). (**B**, **F**, **G**, **I**, **M**, and **N**) 5 HPFs per area were quantified for each mouse. Small gray dots indicate individual HPFs, and black triangles indicate mouse-level means used for statistical analysis. Scale bars: 40 μm. 2-tailed Student’s *t* tests. *P < 0.05; ** *P* < 0.01; *** *P* < 0.001; ****P < 0.0001.

We previously screened a chemical library and identified Am80 (tamibarotene), a synthetic retinoid that has been clinically used as a therapeutic agent for acute promyelocytic leukemia (27), as a reagent that effectively induces Meflin expression in fibroblasts (14, 15, 28). The functional doses and potential cytotoxic effects were evaluated previously (14). Since Meflin expression peaked at the early stage of fibrogenesis, approximately 6 weeks after DSS induction (Figure 2D), we first investigated the effect of the pharmacological induction of Meflin expression by orally administering Am80 to mice subjected to DSS for 7–9 weeks (days 43–63) (Figure 4A). Am80-mediated significant induction of Meflin expression was confirmed by ISH (Figure 4B). Unexpectedly, mice treated with Am80 exhibited a continuous decrease in body weight (Figure 4C). Additionally, gross observation of the colon and histopathological evaluation with H&E staining revealed that the Am80 group exhibited more severe intestinal inflammation, suggesting that Am80 administration exacerbated intestinal inflammation (Figure 4, D and E). However, intestinal fibrosis was significantly ameliorated by Am80 administration, as shown by decreases in the number of α-SMA^+^ myofibroblasts and collagen deposition (Figure 4, F and G).

To explore the optimal timing of Am80 administration to maximize its beneficial therapeutic effect, we next administered Am80 during weeks 10–13, corresponding with the phase in which acute inflammation resolves in the DSS-induced colitis model (Figure 4H) (24). As with the previous dosing regimen (Figure 4A), significant upregulation of Meflin expression was achieved using this regimen (Figure 4I). However, no significant differences in inflammatory indicators, including body weight changes, colon length, or histopathological assessments, were observed between Am80-treated and control groups (Figure 4, J–L). Importantly, the Am80-treated group showed significant mitigation of intestinal fibrosis, as indicated by decreases in the number of α-SMA^+^ myofibroblasts, submucosal collagen deposition, and fibrosis scoring (Figure 4, M and N). These histological findings were further supported by significant downregulation of fibrotic marker genes, including *Acta2, Col1a1, Col3a1,* and *Fn1*, in Am80-treated mice (Supplemental Figure 4, A and B).

We next administered Am80 to the DSS-induced colitis model through the early to fibrotic phase (days 1–42) to test its preventive effect on disease progression (Supplemental Figure 5A). No significant differences in the inflammatory indicators, including body weight changes, colon length, and histopathological assessments, were observed between the Am80-treated and control groups (Supplemental Figure 5, B–D). Interestingly, Am80 administration from the start of DSS induction significantly attenuated the development of intestinal fibrosis, as demonstrated by reduced fibrotic area and collagen deposition at a later time point (week 9) (Supplemental Figure 5, E–G). These data suggest that there are specific therapeutic windows and dosing regimens in which Am80 can be used to suppress, resolve, or even prevent intestinal fibrosis in a DSS-induced colitis model.

Given that the established therapy for patients with IBD has been based on the antiinflammatory effect of the antitumor necrosis factor α (TNF-α) antibody, we next evaluated the synergistic effect of the combination of Am80 and anti–TNF-α antibody on the disease state of the DSS model and examined whether the aggravative effect of Am80 observed in the earlier experiment, shown in Figure 4, A–G, was rescued by the anti–TNF-α antibody (Supplemental Figure 6A). Coadministration of the anti–TNF-α antibody with Am80 did not ameliorate the excessive inflammation induced by Am80, as indicated by comparable body weights, colon length, and histological inflammation (Supplemental Figure 6, B-D). However, the fibrotic reaction was significantly reduced by Am80 administration, regardless of its combination with the anti–TNF-α antibody. Meanwhile, anti–TNF-α monotherapy failed to suppress myofibroblast activation or collagen deposition (Supplemental Figure 6, E–H). The expression levels of inflammatory cytokines, including *Il6*, *Tnf*, and *Il10,* were not significantly different between the Am80 monotherapy and the Am80/anti–TNF-α antibody combination groups (Supplemental Figure 6I).

**Figure 5:**
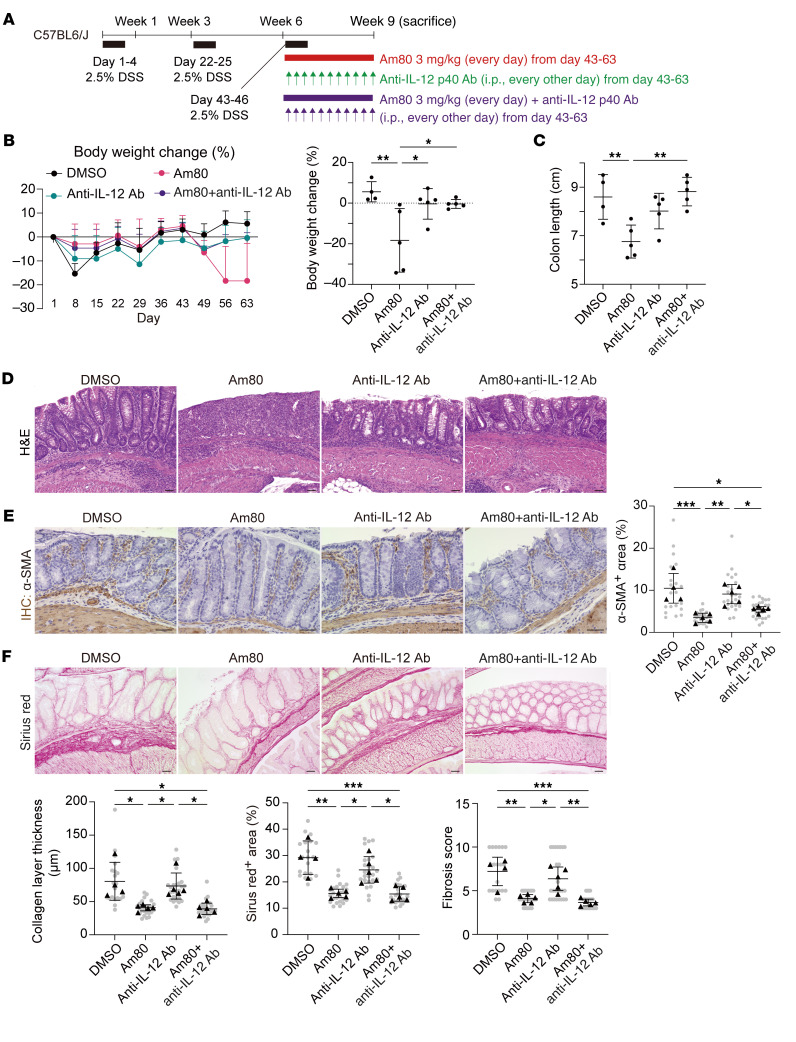
Combined administration of Am80 and anti–IL-12 p40 antibodies attenuates intestinal fibrosis and suppresses Am80-associated intestinal inflammation. (**A**) Schematic diagram showing the experimental setup. Oral administration of Am80 (3 mg/kg/day, every day), anti–IL-12 p40 antibodies (every other day), or the combination of Am80 and anti–IL-12 p40 antibody administration was initiated simultaneously with the 3rd dose of DSS administration from days 43–63. (**B** and **C**) Body weight changes over time and colon length at Day 63 are measured, followed by quantification. (**D**–**F**) Colon tissue sections of mice of the indicated groups are stained with H&E (**D**), examined for α-SMA by IHC (**E**), and stained by Sirius Red (**F**), followed by the quantification of collagen layer thickness, Sirius Red^+^ areas, and fibrosis score (*n* = 4 for control and *n* = 5 for Am80, anti-IL-12 p40 Ab, and Am80/anti–IL-12 combination groups). (**B** right panel, **C**) Each dot represents an individual sample. (**E** and **F**) 5 HPFs per area were quantified for each mouse. Small gray dots indicate individual HPFs, and black triangles indicate mouse-level means used for statistical analysis. Scale bars: 40 μm. One-way ANOVA. *P < 0.05; **P < 0.01; ***P < 0.001; ****P < 0.0001.

Next, we evaluated the therapeutic potential of combining Am80 with an anti–IL-12/23 p40 antibody, which is widely used in clinical practice, particularly for patients with CD who are refractory to anti–TNF-α therapy (29, 30). We coadministered an anti–IL-12 p40 antibody with Am80 in the DSS-induced colitis model (Figure 5A). Interestingly, the coadministration of an anti–IL-12 p40 antibody with Am80 ameliorated the excessive inflammation induced by Am80, as indicated by improved body weight loss, restored colon length, and reduced histological inflammation when compared with mice treated with Am80 alone (Figure 5, B–D). Moreover, the fibrotic reaction was significantly reduced by Am80 administration, regardless of its combination with the anti–IL-12 p40 antibody (Figure 5, E and F). Collectively, these findings indicate that the anti–IL-12 p40 antibody effectively mitigates Am80-associated intestinal inflammation while preserving Am80’s antifibrotic effects, thereby broadening the therapeutic window of Am80 when used in combination with clinically relevant antiinflammatory therapy.

### Am80-induced reversal of intestinal fibrosis is mediated by Meflin expression.

**Figure 6:**
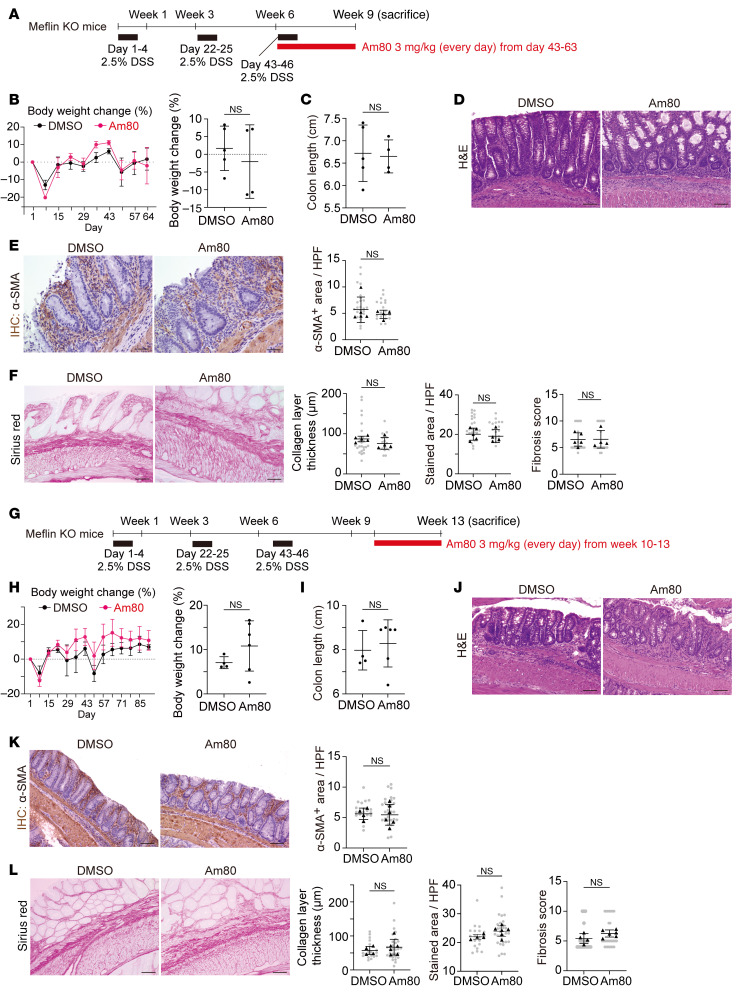
Am80-induced reversion of established intestinal fibrosis is mediated by Meflin expression. (**A**) Schematic diagram showing the experimental setup. (**B** and **C**) Body weight changes over time, and colon length at Day 63 is measured, followed by quantification. (**D**–**F**) Colon tissue sections from Meflin-KO mice after Am80 administration are stained with H&E (**D**), IHC for α-SMA (**E**), and Sirius Red (**F**), followed by collagen layer thickness, quantification of Sirius Red^+^ areas, and fibrosis score. (**E** and **F**) (*n* = 5 and 4 for the control and Am80 groups, respectively). (**G**) Am80 (3 mg/kg/day) or DMSO is orally administered every day to Meflin-KO mice from weeks 10–13 after the 3 cycles of DSS administration. (**H** and **I**) Body weight changes over time, and colon length at Day 92 is evaluated. (**J**–**L**) Representative images for H&E, IHC, for α-SMA, and Sirius Red staining performed in colon tissues from Meflin-KO mice after DSS and subsequent Am80 administrations (*n* = 4 and 6 for the control and Am80 groups, respectively), followed by quantification. (**B** right panel, **C**, **H** right panel, **I**) Each dot represents an individual sample. (**E**, **F**, **K**, **L**) 5 HPFs per area were quantified for each mouse. Small gray dots indicate individual HPFs, and black triangles indicate mouse-level means used for statistical analysis. Scale bars: 40 μm. Statistical significance was determined using 2-tailed Student’s *t* tests.

Given that Am80 is a synthetic retinoid, it may exert many effects on several types of cells besides the induction of Meflin expression in fibroblasts. We next subjected Meflin-KO mice to the DSS-induced colitis model, followed by treatment with Am80 using the same protocols for WT mice (Figure 6A). Meflin-KO mice administered Am80 from days 43–63 exhibited comparable body weight, colon length, and intestinal inflammation to those of the control groups (Figure 6, B–D). No significant difference in fibrotic response was observed between the Am80-treated and control groups (Figure 6, E and F). These results suggest that both the hyperactivated intestinal inflammation and reduced fibrosis induced by Am80 are dependent on Meflin expression.

We then examined whether the efficacy of Am80 in mitigating intestinal fibrosis after the resolution of acute inflammation also depends on the expression of Meflin. Meflin-KO mice subjected to DSS were orally administered Am80 after fibrosis was established for 10–13 weeks (Figure 6G). We did not observe any significant differences in body weight, colon length, inflammatory reaction, number of α-SMA^+^ myofibroblasts, collagen deposition, or fibrosis scores between Am80-treated and control mice (Figure 6, H–L). Moreover, no significant difference was found in fibrotic and inflammatory gene expression in RT-qPCR analysis (Supplemental Figure 7, A and B). Our results indicate that the efficacy of Am80 in suppressing the fibrotic response and reversing established intestinal fibrosis is mediated by Meflin expression induced by Am80 in the DSS colitis model.

### Am80 reverses fibrotic response in cultured fibroblasts and tissue pieces harvested from intestinal stenotic regions of patients with CD.

**Figure 7:**
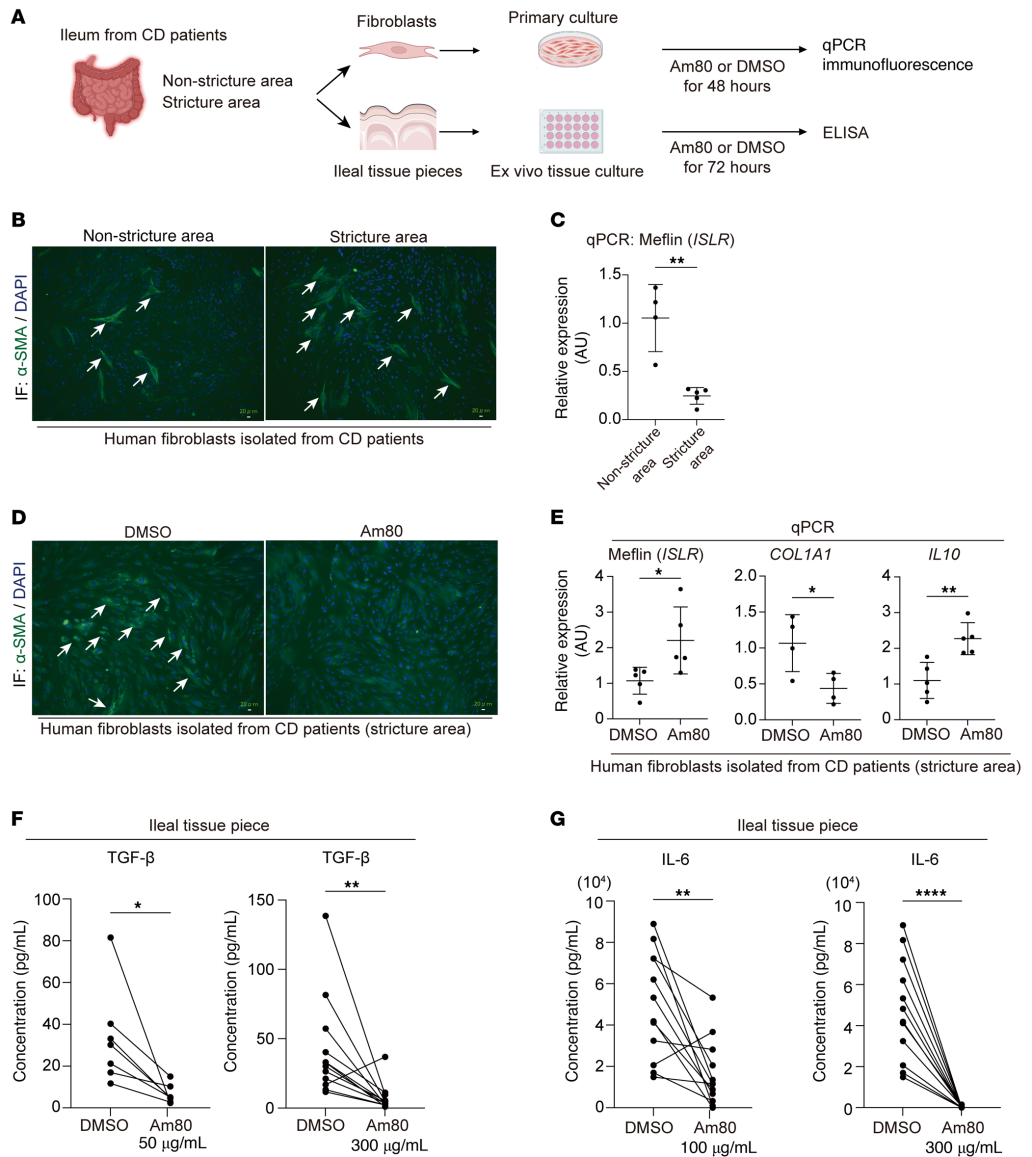
Am80 administration reverses fibrotic response of fibroblasts and tissue pieces isolated from patients with CD. (**A**) Intestinal fibroblasts and ileal tissue pieces are isolated from the intestinal nonstenotic and stenotic areas surgically resected from patients with CD, followed by culture on plastic. These samples are treated with Am80 (50, 100, 300 μg/mL) or DMSO for 48 or 72 hours, followed by the indicated experiments. (**B**) Primary cultured intestinal fibroblasts from the nonstenotic (left) and stenotic (right) areas are fixed and stained for α-SMA using IF. Arrows denote fibroblasts that are strongly positive for α-SMA. Scale bars: 20 μm. (**C**) qPCR analysis for Meflin mRNA expression in the primary cultured intestinal fibroblasts (*n* = 4 and 5 for the nonstenotic and stenotic groups, respectively). (**D**) Intestinal fibroblasts cultured in the absence (left) or presence (right) of Am80 are fixed and subjected to IF for α-SMA. Arrows denote fibroblasts that are strongly positive for α-SMA. Scale bars: 20 μm. (**E**) qPCR analysis for the expression of the indicated genes in the intestinal fibroblasts cultured in the absence (left) or presence (right) of Am80 (*n* = 5 for each group). (**F** and **G**) Ileal tissue pieces obtained from the stenotic regions of intestine are incubated with different concentrations of Am80 (50 and 300 μg/mL) or DSMO for 24 hours, followed by the measurement of TGF-β (**F**) and IL-6 (**G**) in the culture supernatants using enzyme-linked immunosorbent assays (*n* = 7 to 12 for each group). (**C**, **E**, **F**, **G**) Statistical significance was determined using 2-tailed Student’s *t* tests. *P < 0.05; **P < 0.01; ****P < 0.0001.

To test whether Am80 could be used to treat patients with CD, we isolated intestinal fibroblasts from both nonstricture and the stricture areas of surgically resected intestines of patients with CD and cultured them in the presence or absence of Am80, followed by gene expression analysis (Figure 7A). Immunofluorescence (IF) staining showed more prominent expression of α-SMA in intestinal fibroblasts isolated from stricture areas than in those isolated from nonstricture areas (Figure 7B). We also confirmed that Meflin expression was significantly downregulated in intestinal fibroblasts from stricture areas compared with those from nonstricture areas (Figure 7C). The treatment of fibroblasts isolated from the stricture area with Am80 for 48 hours resulted in a significant decrease in α-SMA expression (Figure 7D), which was accompanied by the upregulation of expressions of Meflin and *IL-10* and downregulation of collagen type I (*COL1A1*) expression (Figure 7E).

To further test the antifibrotic effect of Am80 in a model that recapitulates human intestinal fibrosis, we prepared ileal tissue pieces from surgically resected specimens of 7 patients with CD and put them in ex vivo tissue culture for 72 hours either in the presence or absence of different doses of Am80 (50–300 μg/mL) in the medium (Figure 7A). The measurement of the secretion of profibrotic cytokines using ELISA showed that the amounts of active and latent form of TGF-β and IL-6 were significantly reduced by Am80 treatment (Figure 7, F and G), suggesting that Am80 may be potent in inhibiting the secretion of profibrotic cytokines and ameliorating established intestinal fibrosis in patients with CD.

### Meflin deficiency leads to the upregulation of the noncanonical Wnt ligand WNT5A and its receptor ROR2.

**Figure 8:**
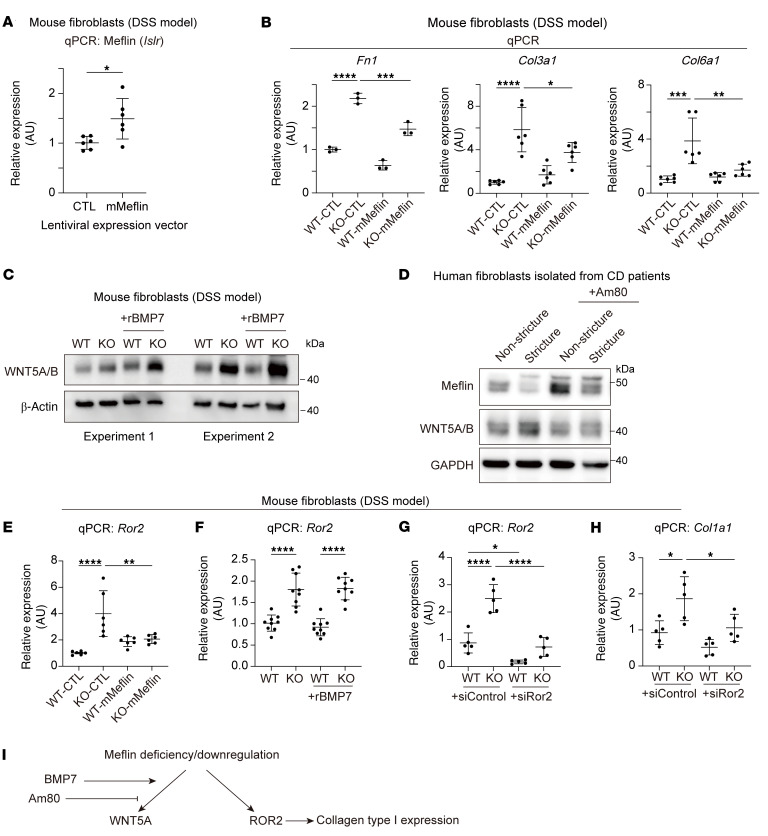
Meflin may inhibit intestinal fibrosis by suppressing the expression of WNT5A and its receptor ROR2. (**A**) Colonic fibroblasts isolated from WT mice after 9 weeks of DSS administration were transduced with control or mouse Meflin (mMeflin) cDNA using a lentiviral expression system, followed by qPCR analysis (*n* = 3 per group). CTL, control. 2-tailed Student’s *t* tests. (**B**) qPCR analysis for the expression of *Fn1, Col3a1,* and *Col6a1* in the indicated fibroblasts (*n* = 3–6 per group). (**C**) Colonic fibroblasts isolated from WT and Meflin-KO mice after 9 weeks of DSS administration were cultured with or without recombinant BMP7 (rBMP7, 100 ng/mL) for 1 hour, followed by Western blot analysis for WNT5A/B and β-actin. The data from 2 independent experimental batches are shown. kDa, kilodaltons. (**D**) Colonic fibroblasts isolated from nonstricture and stricture areas of patients with CD were treated with Am80 (1 μM) for 48 hours, followed by Western blot analysis for Meflin, WNT5A/B, and GAPDH. (**E**) qPCR analysis for *Ror2* mRNA expression in colonic fibroblasts isolated from WT and Meflin-KO mice. Restoring Meflin expression (mMeflin) in Meflin-KO fibroblasts significantly suppressed Ror2 expression (*n* = 6 per group). (**F**) Colonic fibroblasts isolated from WT and Meflin-KO mice were treated with rBMP7 (20 ng/ml) for 48 hours, followed by qPCR analysis for *Ror2* mRNA expression (*n* = 9 per group). (**G** and **H**) Colonic fibroblasts were transfected with control siRNA (siControl) or siRor2, followed by qPCR analysis for *Ror2* (**G**) in *Col1a1* (**H**) mRNA levels (*n* = 3 per group). (**I**) Summary of the findings obtained by colonic fibroblasts from WT and Meflin-KO mice and Ror2 knockdown. (**B**, **E**, **F**) Each dot represents an individual sample. Scale bars: 40 μm. 1-way ANOVA unless otherwise indicated. *P < 0.05; **P < 0.01; ***P < 0.001; ****P < 0.0001.

Given the role of Meflin in intestinal fibrogenesis, we sought to elucidate the underlying mechanisms. We isolated colonic fibroblasts from both WT and Meflin-KO mice after DSS administration (Supplemental Figure 8A) and transduced those fibroblasts with mouse Meflin (mMeflin) cDNA using a lentiviral expression system (Figure 8A). Notably, the elevated expression of fibrotic markers, including *Fn1, Col3a1,* and *Col6a1*, observed in Meflin-KO fibroblasts was significantly reversed by mMeflin restoration (Figure 8B). These results confirmed that Meflin may confer an antifibrotic role to fibroblasts. To further characterize Meflin^+^ fibroblasts, we analyzed a publicly available single-cell RNA-seq dataset from NCBI Gene Expression Omnibus (GEO; GSE211275) for mouse and human colonic mesenchymal cells (8). The analysis revealed that Meflin and various BMP signaling components are highly coexpressed within several intestinal mesenchymal cell populations across the species (Supplemental Figure 8, B–F, and Supplemental Tables 2 and 3), consistent with our previous studies, which showed that Meflin augments BMP7 signaling in fibroblasts (12, 16). However, Meflin deficiency or exogenous mMeflin transduction had little-to-no effect on BMP7-mediated SMAD1/5/9 phosphorylation, cell proliferation, or the expression of collagen genes (*Col1a1* and *Col3a1*) (Supplemental Figure 8, G–I). Basal Smad1/5/9 phosphorylation and the proliferation rate of Meflin-KO fibroblasts were lower and higher, respectively, compared with WT fibroblasts, and these differences were negated by BMP7 stimulation, which is of unknown significance at present (Supplemental Figure 8, G and H). These data showed the limited functional involvement of Meflin in BMP7/SMAD signaling at least in intestinal fibroblasts.

We next sought to identify other changes in gene expression associated with Meflin. Gene ontology enrichment analysis of high–Meflin-expressing cell clusters identified from scRNA-seq datasets of intestinal tissues from patients with CD (GSE134809) and from the DSS-induced colitis model (GSE172261) showed an enrichment of genes associated with Wnt signaling (Supplemental Figure 9A). Further analysis of both datasets revealed that Meflin is coexpressed with the noncanonical Wnt ligand *Wnt5a* in some intestinal stromal populations (Supplemental Figure 9, B-E). Our analysis also demonstrated that Meflin^+^ cells are distinct from CDH11^+^ cells, which have been reported to promote intestinal fibrosis (8) (Supplemental Figure 9F). Considering a recent report that fibroblasts positive for *WNT5A* are highly enriched in the strictured lesions to play a central role in fibrogenesis in patients with CD (8), we hypothesized that Meflin suppresses the fibrogenesis by regulating WNT5A expression.

We observed an upregulation of WNT5A expression in Meflin-KO fibroblasts and human intestinal fibroblasts isolated from the stricture area of the ileum of patients with CD, and this was modestly promoted by recombinant BMP7 (rBMP7) stimulation and suppressed by Am80 treatment, consistent with previous reports demonstrating that retinoic acid signaling counteracts Wnt signaling (31, 32) (Figure 8, C and D). Meflin KO fibroblasts exhibited an upregulation of the WNT5A receptor *Ror2* (33, 34), which was negated by mMeflin restoration but independent of rBMP7 stimulation (Figure 8, E and F). Importantly, siRNA-mediated knockdown of *Ror2* in Meflin-KO fibroblasts significantly reduced *Col1a1* expression (Figure 8, G and H). These data suggested that Meflin deficiency in fibroblasts is associated with increases in the expression of WNT5A and its receptor ROR2 that induce fibrogenesis, which could be modulated by BMP7 and Am80 (Figure 8I).

### Meflin deficiency is associated with Wnt signaling dysregulation in the DSS-induced colitis model.

**Figure 9:**
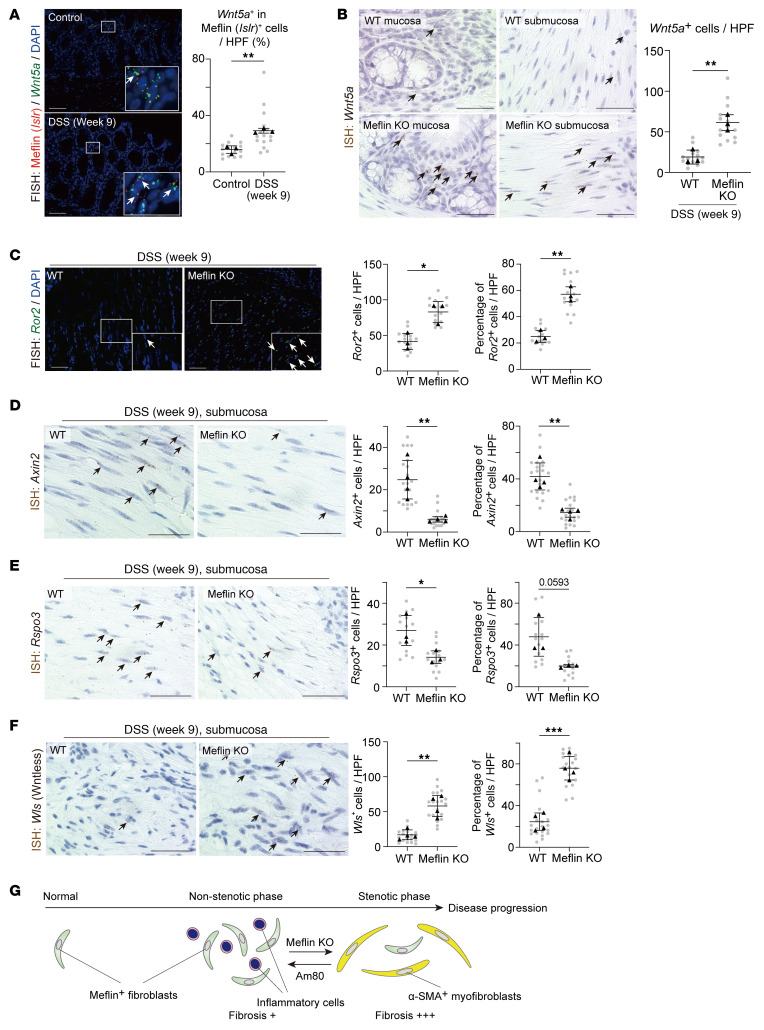
Dysregulated Wnt signaling in Meflin-deficient mice. (**A**) Colon tissue sections prepared from intestines of WT mice treated with DSS are stained for Meflin (*Islr*, red) and *Wnt5a* (green) mRNA by FISH (left), followed by quantification of the positivity of *Wnt5a* in Meflin^+^ fibroblasts (arrows) (right). Boxed areas are magnified in insets (*n* = 3 per group). (**B**–**F**) Colon tissue sections prepared from the intestines of WT and Meflin-KO mice treated with DSS are stained for *Wnt5a* (**B**), *Ror2* (**C**), *Axin2* (**D**), *Rspo3* (**E**), and *Wls* (**F**) using ISH or FISH (left panels), followed by quantification of the numbers of positive cells and percentage of those cells per all stromal cells with a fibroblast-like morphology (right panels). (*n* = 3–4 for each group). Arrows indicate the positive cells. (**G**) Schematic illustration showing the hypothesis proposed by the present study. (**A**–**F**) 5 HPFs per area were quantified for each mouse. Small gray dots indicate individual HPFs, and black triangles indicate mouse-level means used for statistical analysis. Scale bars: 40 μm. Statistical significance was determined using 2-tailed Student’s *t* tests. *P < 0.05; **P < 0.01; ***P < 0.001.

Finally, we examined the dysregulation of WNT5A and ROR2 expression in tissue samples of the DSS-induced colitis model. ISH analysis showed that the expression of *Wnt5a* in Meflin^+^ fibroblasts was more evident in mice undergoing DSS-induced chronic inflammation than in control mice (Figure 9A). The number of Wnt5a^+^ fibroblasts was higher in Meflin-KO mice treated with DSS than in WT mice (Figure 9B). In contrast, the number of Meflin^+^ fibroblasts positive for *Wnt2b*, a canonical Wnt ligand (35), was reduced in the DSS model (Supplemental Figure 10A), and *Wnt2b* expression in fibroblasts was significantly downregulated in Meflin-KO mice subjected to the DSS model (Supplemental Figure 10B). Consistent with the results that showed the high expression of *Ror2* in intestinal fibroblasts isolated from Meflin KO mice (Figure 8, E and F), ISH analysis confirmed the upregulation of *Ror2* in the intestinal fibroblasts of Meflin-KO mice after DSS treatment, suggesting activation of the noncanonical Wnt pathway in Meflin-KO mice (Figure 9C). Conversely, the expression of the canonical Wnt target genes *Axin2* and R-spondin 3 (*Rspo3*) in fibroblasts was attenuated in Meflin-KO mice compared with that in WT mice (Figure 9, D and E). We also found the compensatory upregulation of Wntless (encoded by the *Wls* gene), which plays an indispensable role in the transportation and secretion of Wnt ligands (36), in fibroblasts of Meflin-KO mice treated with DSS (Figure 9F, Supplemental Figure 10C).

Collectively, these data suggest that Meflin deficiency is associated with the upregulated production of noncanonical Wnt ligands and their activation, thus contributing to the activation of intestinal fibrogenesis.

## Discussion

The development of antifibrotic drugs for patients with IBD lags behind that of other organ diseases (37). Recent milestone studies have demonstrated potential therapeutic effects on intestinal fibrosis (21–23). However, these studies have primarily focused on targeting liquid factors, such as cytokines and chemokines, or components of the extracellular matrix, and the fundamental role of fibroblast heterogeneity in the etiology of intestinal fibrosis remains an issue in this field. Our study showed that Meflin^+^ fibroblasts comprise a functional fibroblast subset that plays a key role in suppressing intestinal fibrogenesis by regulating the expression of the noncanonical Wnt regulators WNT5A and ROR2. We also showed that pharmacological manipulation of Meflin expression in fibroblasts could reduce the proportion of α-SMA^+^ myofibroblasts and reverse the established fibrosis of the intestines found in CD (Figure 9G).

We demonstrated that Meflin expression in fibroblasts was significantly reduced in the stricture areas of the intestines of patients with CD and established fibrosis in colitis mouse models induced by DSS administration or IL-10 deficiency. Notably, the number of Meflin^+^ fibroblasts increased in the early stages of fibrosis, followed by a decrease in the later stages of the DSS model. Previous studies have shown that the loss of Meflin expression is related to greater vulnerability to cardiac fibrosis, myocardial infarction, and acute lung injury in respective mouse models (10, 38). This notion is supported by a previous study, which showed that specific deletion of the Meflin gene in Twist2^+^ stromal cells impaired intestinal regeneration in the DSS model (10). Thus, the primary role of Meflin may be both the repair and regeneration of acutely injured tissues and suppression of the fibrotic response in the chronic phases of diseases. Measuring the Meflin levels in the biopsy samples may allow monitoring of the disease state of patients with CD who usually show cyclical exacerbation with disease relapse and remission or detect those with early-stage fibrosis before the appearance of clinical symptoms (38).

We further demonstrated the potential therapeutic effects of Am80 administration against intestinal fibrosis (Figure 9G). These results suggest that the modulation or control of fibroblast heterogeneity could be an approach for treating intestinal fibrosis in patients with IBD, including CD. However, a limitation is that Am80 administration in the early CD phase may aggravate inflammatory flares rather than suppress fibrosis. Importantly, we found that this Am80-induced inflammatory response was effectively rescued by the coadministration of anti–IL-12p40 — but not anti–TNF-α — antibodies. These findings indicate that the inflammatory side effects of Am80 are primarily mediated through the activation of helper T cells, rather than of other immune cells, which activate TNF-α, including dendritic cells (39), although the precise immune mechanisms remain to be elucidated. This observation broadens the potential therapeutic scope of Am80. Therefore, the combination of Am80 and anti–IL-12/23 biologics, such as ustekinumab (40), may represent a rational therapeutic strategy for managing both inflammation and fibrosis in treating patients with CD. Alternatively, patients with CD in the remission phase or in good control of their inflammation may benefit from Am80 administration. Another potential limitation of Am80 is that the unknown implications for the epithelial homeostasis and immune system as an artificial compound. Further research is necessary to delve into the detailed mechanisms by which Am80-mediated control of fibroblast heterogeneity spatially and temporally alters intestinal inflammation and fibrosis before translating these findings into clinical settings.

The present study revealed that the role of Meflin in intestinal fibroblasts may be distinct from that in fibroblasts isolated from other organs, showing a minor and negligible role in BMP7/Smad signaling. We observed the upregulation of the noncanonical Wnt ligand WNT5A and its receptor *Ror2* in Meflin-KO fibroblasts, which was accompanied by the downregulation of the canonical Wnt signaling. This was consistent with previous studies that showed that the WNT5A-ROR2 signaling promotes fibroblast activation and proliferation during tissue regeneration and remodeling (41, 42). Our functional rescue experiment demonstrated that Meflin significantly suppressed the expression of *Ror2* and genes related to fibrogenesis. From a mechanistic perspective, the GPI-anchored membrane or extracellular localization of Meflin (9) suggests that it suppresses WNT5A and ROR2 upregulation by interacting with other ligands or cell-surface receptors. Further studies are required to reveal how Meflin constrains fibroblast activation by limiting the expression of WNT5A and ROR2.

Another limitation of this study is that the chronic DSS-induced colitis model does not fully capture the formation of luminal strictures observed in patients with CD. The pathogenesis of CD involves complex interactions among genetic susceptibility, environmental factors, and dysregulated immune responses, making faithful modeling of progressive structural changes in mice inherently challenging. Future studies employing surgically induced stricture models, patient-derived organoid-on-a-chip systems, and lineage-specific conditional knockout approaches targeting Wnt-related signaling components within the Meflin lineage will be important for validating the mechanistic framework proposed in this study.

In summary, Meflin expression in a fibroblast subset suppresses intestinal fibrosis by regulating Wnt signaling. Pharmacological induction of Meflin expression alleviates intestinal fibrosis in a mouse model of colitis and in tissue samples from patients with CD. Thus, Meflin^+^ fibroblasts may represent a therapeutic target for intestinal fibrosis in patients with CD.

## Methods

### Sex as a biological variable.

Our study exclusively examined male mice. It is unknown whether the results would be similar in female mice, although we would not expect significant differences in the results. Human samples were obtained from both male and female participants.

### Human samples.

Freshly resected intestinal specimens from patients with CD were obtained from Nagoya University Hospital.

Briefly, the presence of luminal strictures was mapped using endoscopy and/or cross-sectional imaging in patients with CD before resection and confirmed by the surgeon. After resection, matched samples from the stricture and stricture-adjacent but non-stricture regions were obtained from the same CD specimens, followed by the preparation of formalin-fixed paraffin-embedded samples. Freshly resected samples from the respective regions were preserved in RNAlater preservative for later RNA isolation and RT-qPCR. For the isolation of intestinal fibroblasts and *ex vivo* culturing of the ileal tissue pieces, fresh samples were kept in ice-cold Dulbecco’s phosphate-buffered saline (D-PBS; Wako, Fujifilm, Japan) or RPMI1640 (Wako) with penicillin-streptomycin (Gibco, Life Technologies, CA) and transported to the laboratory immediately for the following steps.

### Mice.

Meflin-KO mice were previously generated in our laboratory (9, 12–14). IL-10–KO mice (B6.129P2-Il10tm1Cgn/J, strain 002251, Jackson Laboratory) were purchased and used as a mouse model of colitis that spontaneously develops intestinal inflammation and fibrosis (43). To generate double-KO mice (Meflin/IL-10 DKO), Meflin-KO mice were crossed with *Il10* heterozygous mice (Il10^-/+^), and littermates that were double heterozygous for both *Islr* and *Il10* (*Islr*^-/+^*Il10*^-/+^) were crossed to obtain Meflin/IL-10 DKO mice. C57BL/6J WT mice were purchased from Charles River Laboratories Japan, Tokyo, Japan. All mice were maintained on a C57BL/6J background, with 8- to 10-week-old males used in this study. All mice were maintained under specific pathogen-free conditions with a 12-h day/night cycle and controlled humidity (40%–60%) and temperature (18–23°C), and housed and kept in ventilated cages with standard bedding and had free access to sterile drinking water and mouse food *ad libitum* to minimize any potential effect of different microbiota. Mice were randomly assigned to groups. Animal experiments were approved by the Animal Care and Use Committee of Nagoya University Graduate School of Medicine (approval number M240024) and performed in compliance with the regulations and guidelines of animal care and use of Nagoya University.

### Am80 administration experiments.

For in vivo experiments to test the effect of Am80 treatment, 8-week-old male WT or Meflin-KO mice subjected to the DSS-induced chronic intestinal fibrosis model were administered Am80 (3 mg/kg/day, Tocris Bioscience, UK) dissolved in 0.5% carboxymethylcellulose solution (Wako Chemicals, Osaka, Japan) by oral gavage for the indicated periods. An equal volume of dimethyl sulfoxide (DMSO, Wako) was used as a control. In the experiment to test the effect of a combination of Am80 and anti–TNF-α antibodies, 10 mg/kg InVivoMAb anti-mouse TNFα antibodies (Clone TN3-19.12, Bioxcell, USA) were intraperitoneally injected three times per week from the 7th to the 9th week (day 43–63). To evaluate the effects of combined treatment of Am80 and anti–IL-12 p40 antibodies, mice received intraperitoneal injections of InVivoMAb anti-mouse IL-12 p40 antibodies (10 mg/kg; #BE0051, Bio X Cell, USA) every other day from week 7 to week 9 (day 43–63). All mice were sacrificed on the day following the final administration of Am80 or vehicle.

For investigating the treatment effect of Am80 on fibroblasts of patients with CD, primary cultured intestinal fibroblasts isolated from the stenotic and non-stenotic regions of the resected samples were cultured on 6-cm^2^ plates and treated with Am80 (1 μM) or DMSO (1 μM) after reaching 100% confluent. After a 48-h culture, the cells were harvested, and total RNA and protein were extracted for RT-qPCR and western blot analyses.

### Lentiviral production and transduction.

For the induction of Meflin expression, a lentiviral system was utilized. On day 0, HEK293 cells were seeded at a density of 2.0 × 10^6^ cells onto collagen-coated 10-cm dishes. On the following day, the cells were transfected with a DNA-Lipofectamine complex consisting of 4 μg of the gene expression vector, either the pLV-CMV-Ash-MCL-Puromycin vector (pLV-mMeflin) (Medical & Biological Laboratories Co., Ltd, Nagoya, Japan) or pLV-CMV-Azami-Green-MCL-Puromycin vector (pLV-CTL) (Medical & Biological Laboratories 14 Co., Ltd, Nagoya, Japan), 12 μg of lentiviral packaging vectors (Thermo Fisher Scientific, Waltham, MA), and 20 μL of Lipofectamine 2000 (Thermo Fisher Scientific, Waltham, MA) diluted in Opti-MEM. After 4 h of incubation at 37°C, the transfection medium was replaced with low-glucose DMEM supplemented with 10% fetal bovine serum. On day 3, the lentivirus-containing culture supernatant was harvested, centrifuged at 3,000 rpm for 10 min, and passed through a 0.22 μm filter. The target fibroblasts were then incubated with this filtered viral supernatant at 37°C. Stable Meflin-expressing cells were selected by culturing the cells in medium containing 1 μg/mL puromycin once they reached 70-80% confluence.

For rBMP treatment, mouse primary intestinal fibroblasts at approximately 90% confluence were seeded and serum-starved for 12 h, followed by stimulation with rBMP7 (100 ng/ml, R&D system, 5666-BP) for 1 h, after which cells were harvested according to the protocol described below. Cells were cultured with rBMP7 (20 ng/ml) for 48 h without serum starvation for RT-qPCR analysis.

### siRNA-mediated Ror2 knockdown.

For Ror2 knockdown, primary mouse intestinal fibroblasts were transfected with either a negative control siRNA (Thermo Fisher Scientific, Pleasanton, CA) or siRor2 (s77264, Thermo Fisher Scientific, Pleasanton, CA) using Lipofectamine RNAiMAX Reagent (Thermo Fisher Scientific, Pleasanton, CA). Transfections were performed in a 6-well plate format. Briefly, cells were seeded to reach approximately 80% confluence at the time of transfection. For each well, 30 pmol of siRNA was diluted in Opti-MEM, while 5 μL of Lipofectamine RNAiMAX was diluted separately in Opti-MEM. The diluted siRNA and Lipofectamine RNAiMAX solutions were then combined and incubated for 20 min to allow formation of RNA-lipid complexes. These complexes were added dropwise to cells cultured in antibiotic-free medium. After 5 h of incubation at 37°C in a 5% CO_2_ atmosphere, the transfection medium was replaced with fresh culture medium. Knockdown efficiency was assessed at 24, 48, 72, and 96 h after transfection.

### Statistics.

Data were presented as individual data points. Mean ± SD are presented in all figure parts in which error bars are shown. For comparisons between two groups, paired or 2-tailed Student’s *t* tests were used, as appropriate. For comparisons among multiple groups, 1-way ANOVA was used. Statistical analyses were performed using GraphPad Prism version 9 (GraphPad Software). A *P* value less than 0.05 was considered significant.

Detailed analysis protocols are described in the Supplemental Data.

### Study approval.

All samples were obtained from patients who provided written, informed consent. The patient study was conducted in accordance with the principles of the Declaration of Helsinki and approved by the ethics committee of Nagoya University Graduate School of Medicine (Nagoya, Japan, approval ID: M230086). The animal experiments were approved by the Institutional Animal Care and Use Committee of Nagoya University. (Nagoya, Japan; no. M240024) and were carried out following the Institutional Ethical Guidelines for Experiments with Animals, as well as the Guide for the Care and Use of Laboratory Animals.

### Data availability.

All data supporting the findings of this study are available within the article and its supplemental material. Supporting Data Values are provided in an XLS file. Additional data is available from the corresponding author upon reasonable request.

## Author contributions

JM and K Maeda designed research studies, conducted experiments, analyzed data, and wrote the manuscript. AO conducted experiments. SM, NE, YS performed histological analyses and provided pathological expertise. YM, TK, HS, and GN provided reagents. TI, MN, TY, TS, EI, K Murate, TH, and KF contributed to experimental design and data interpretation. MF, HK, and AE supervised the study and critically revised the manuscript. All authors reviewed edited and approved the manuscript.

## Conflict of interest

The authors have declared that no conflict of interest exists.

## Funding support

- Japan Science and Technology Agency (JST) grants JPMJMS2214-11 (Moonshot R&D to HK), JPMJSP2125 (SPRING to JM), and JPMJFR245K FOREST (KM).
- Japan Society for the Promotion of Science (JSPS) KAKENHI grants JP21K15995 (YM), JP21K15920 (KM), 22H02848 and 22K18390 (AE).
- Japan Agency for Medical Research and Development (AMED) grants JP24gm1210009 and JP24ama221333 (AE) and JP256f0137010 (AE and KM).
- Takeda Science Foundation (KM).
- JSIBD Grants-in-Aid for IBD Research (KM).

## Supplementary Material

Supplemental data

Unedited blot and gel images

Supporting data values

## Version 1 05/19/2026

In-Press Preview

## Version 2 07/01/2026

Electronic publication
