# Supplementary material for: Meflin confers antifibrotic properties to intestinal fibroblasts in inflammatory bowel disease
Source: J Clin Invest. 2026 May 19;136(13):e192804. doi: 10.1172/JCI192804 (PMC13318116; doi:10.1172/JCI192804)
Supplement: Supplemental data [file jci-136-192804-s217.pdf]

Meflin confers antifibrotic properties to intestinal fibroblasts in inflammatory bowel disease

Mu et al.

Supplemental Information

1. Supplemental Tables 1-3

2. Supplemental Figures and Figure legends 1-10

3. Supplemental Methods

Supplemental Table1. Background and clinical information of Crohn's disease patients from whom ileal biopsy samples were obtained.

	N = 24
Characteristics	
Male, n (%)	16 (67)
Median age, y (range)	55 (30-77)
Median disease duration, y (range)	13.1 (2-26)
Location, n (%)	
L1: Ileal	13 (37)
L3: Ileocolonic	11 (63)
Behavior, n	
B1: Inflammatory	2
B2: Stricturing	22
Median CDAI* (range)	100 (10-283)
Median CRP, mg/L (range)	0.3 (0.01-1.75)
Concomitant treatment, n (%)	
Steroid	0 (0)
Immunomodulators	7 (29)
Biologics	21 (88)

*Crohn's Disease Activity Index

Supplemental Table 2. List of top 30 marker genes for mesenchymal cell clusters of the mouse colon identified by a single-cell RNA sequencing analysis (GSE211275)

Cell cluster	SFRP1_MFAP4_ fibroblasts	ECM-producing fibroblasts	Vascular endothelial cells	DCN_ fibroblasts	BMPs_ mesenchymal cells
1	Serpina3n	mt-Co3	Flt1	Fth1	Aldh1a1
2	Sfrp1	mt-Atp6	Plvap	Gm42418	Ogn
3	Tnfaip6	mt-Co2	Fabp4	AY036118	Tmem158
4	Serpina3g	Col3a1	Pecam1	Tpt1	F3
5	Htra3	C4b	Egfl7	Dcn	Bmp5
6	Sod3	mt-Nd4	Kdr	Rps12	4-Sep
7	Mfap4	mt-Co1	Podxl	Gnb2	Sox6
8	Fam198b	mt-Cytb	Ptprb	Lamp1	Ednra
9	Igfbp5	Col1a1	Esam	Sec62	Gnai1
10	Hsd11b1	Gsn	Cd93	Serbp1	Pdlim3
11	Fgfr2	mt-Nd2	Apold1	Mtch1	Nbl1
12	Edil3	mt-Nd1	Slco2a1	Srsf3	Emid1
13	Igfbp4	Malat1	Srgn	Atp5a1	Bmp3
14	Bgn	Col1a2	Adgrf5	Eif4a1	Rerg
15	Dcn	Col5a1	Ehd4	Eif4a2	Fgf9
16	Ccl11	Ltbp4	Cdh5	Tmed10	Bmp7
17	Selenop	Tnfaip2	Cyyr1	Calm2	Parm1
18	Aldh1a3	Meg3	Mmrn2	Calu	Basp1
19	Clec3b	Col15a1	Rasip1	Sh3glb1	Pdgfra
20	Ccdc80	Lrp1	Cd81	Arf1	Wnt5a
21	Vcam1	Fbln1	Slfn5	Pdia6	Col4a5
22	Postn	Col4a1	Ctla2a	Gnb1	Fibin
23	Ncam1	mt-Nd5	Itga6	Sfr1	Rbp4
24	Selenbp1	mt-Nd3	Rgcc	Tmsb4x	Bean1
25	Ltbp1	Neat1	Hspb1	7-Sep	Tmem119
26	Lepr	Col5a2	Tinagl1	Pcbp1	Cybrd1

27	Cygb	Eln	Adgrl4	Cfl1	Alcam
28	Fbln1	Fbln2	Cavin2	Tmed9	Casq2
29	Matn2	Adamts4	Mef2c	Hnrnpk	Hspa12a
30	Gstm1	Tnxb	Emcn	Tpm4	Myl9

Cell cluster	Mito-high fibroblasts	GREM1_ fibroblasts	ADAMDEC1_Inflammatory fibroblasts 2	MYH11_ACTA2_ Smooth muscle cells	Pericytes
1	Malat1	Grem1	Adamdec1	Myh11	Cpe
2	mt-Cytb	Prss23	Zeb2	Actg2	Nrip2
3	mt-Co3	Col14a1	Sfrp1	Hhip	Rgs7bp
4	Tcf4	Gas1	Tmem176a	Lmod1	Itga7
5	mt-Nd1	Slit3	Rarres2	Cnn1	Cox4i2
6	mt-Co2	Igfbp6	Postn	Actc1	Des
7	mt-Atp6	C3	Tmem176b	Frem2	Aoc3
8	mt-Co1	Fmo2	Clec3b	Cdh6	Lmod1
9	mt-Nd4	Ebf1	Efemp1	Dtna	Ttll7
10	mt-Nd2	Aebp1	Serpina3n	Pdgfc	Ebf1
11	Pdgfra	Gsn	Lepr	Myocd	Atp1b2
12	Nrg1	Gfpt2	Hpse	Kcnma1	Itga4
13	Sox6	Tnxb	Lpl	Acta2	Cystm1
14	Runx1	Rbp1	Serping1	Tagln	Rasd1
15	Fosb	Flrt2	Vcan	Tes	Myo1b
16	F3	Adgrd1	Hsd11b1	Myl9	Myh11
17	Col6a4	Hspb8	Igfbp4	Tpm2	Klhl23
18	Btg2	Lum	Itih5	Map1b	Fam241a
19	Gm26532	Cd248	Lgmn	Flna	Pdgfa
20	Col4a5	Dcn	Fam198b	Mylk	Nr2f2
21	mt-Nd3	Svep1	C4b	Dmd	Ntn4
22	Bmp2	Has1	Htra3	Fbxl22	Rcan2
23	Pappa	Cd55	Vstm4	Tpm1	Rasgrp2
24	Pmepa1	Foxp2	Fzd1	Ppp1r12b	Tinagl1
25	Gm26903	Fbn1	Tcf21	Pgm5	Mef2c
26	Bmp5	Ar	Lrp1	Ckb	Pdgfrb

27	Lamb1	Stc2	Ifi27l2a	Des	Gucy1b1
28	Emid1	Dpep1	Col15a1	Pdlim3	Plce1
29	Neat1	Cd81	Fgfr2	Slc24a3	Sncg
30	Gja1	Penk	Dclk1	Lpp	Gja4

Cell cluster	Glial-like mesenchymal cells	Lymphatic endothelial cells	Activated vascular endothelium
1	Plp1	Mmrn1	Selp
2	Kcna1	Ccl21a	Ackr1
3	Kcna2	Reln	Madcam1
4	Ank3	Lyve1	Sele
5	Mal	Flt4	Hdc
6	Cdh19	Lcn2	Cytl1
7	Gpr37l1	Prox1	Adgrg6
8	Ptprz1	Adgrg3	Cldn5
9	Ptn	Gpm6a	Samsn1
10	Gfra3	Cldn5	Aqp1
11	Fcgr2b	Stab1	Vwf
12	Csmd1	Ackr2	Klk8
13	Slc35f1	Aqp1	Lrg1
14	Cadm2	Slc45a3	Csf2rb
15	Foxd3	Dtx1	Meox2
16	S100b	Cyp4b1	AU021092
17	Sox10	Gm21541	Tmem252
18	Kcna6	Mrc1	Rasgef1a
19	Sostdc1	Wipf3	Dusp2
20	Tmprss5	Klhl4	Upp1
21	Gfra1	Sh3gl3	Il2rg
22	L1cam	Sema3d	Traf1
23	Cadm4	Megf6	Slfn2
24	Iqgap2	Gmfg	Rapgef5
25	Plekhb1	Gpr182	Itgb4
26	Chl1	Arhgdib	Rnd1

27	Adam11	Sema3a	Mecom
28	Nkd2	Ptpn18	Ehd3
29	Sema3b	Gpihbp1	Mir155hg
30	Aatk	Kbtbd11	Slco2a1

Supplemental Table 3. List of top 30 marker genes for mesenchymal cell clusters of the colon of patients with Crohn's disease identified by a single-cell RNA sequencing analysis (Mukherjee et al., 2023)

Cell cluster	Fibroblast stem	Endothelial_ venular	Adventitial_ Fibroblast_PI16	Fibroblast_SOD2_ THBS1	Fibroblast_CXCL14_ ADAMDEC1
1	SFRP2	ACKR1	IGFBP6	FOSB	ADAMDEC1
2	MGP	CCL14	MFAP5	IER3	CXCL14
3	CFD	SELE	FBN1	SOD2	CCL11
4	FBLN1	C2CD4B	CLEC3B	THBS1	CTSC
5	DCN	CD74	FSTL1	MEG3	EDIL3
6	GREM1	TM4SF1	CFD	NEAT1	A2M
7	GSN	VWF	TNXB	C3	IGHA1
8	CCDC80	PECAM1	DCN	NR4A1	APOE
9	LUM	NCOA7	PLAC9	EGR1	ADAM28
10	ADH1B	CLDN5	S100A4	LMNA	CXCL1
11	SFRP1	ICAM1	FN1	KDM6B	COL18A1
12	COL14A1	AQP1	SEMA3C	GPRC5A	CXCL6
13	C1R	HLA-DRA	CD248	CEBPB	TCF21
14	IGFBP6	SPARCL1	PI16	MEG8	TCIM
15	C3	HLA-DRB1	EFEMP1	ABL2	NID1
16	GPNMB	CSF3	CD55	DNAJB1	CFH
17	S100A4	HLA-E	IGFBP5	ZFP36L1	JCHAIN
18	RARRES2	CNKSR3	PCOLCE2	CCDC80	GGT5
19	SLIT3	ADAMTS9	FBLN2	GEM	COL3A1
20	PLAC9	LIFR	KRT24	TNFAIP2	IGKC
21	TNXB	ETS2	COL1A2	PIM1	EMILIN1
22	MMP2	IL6	SLPI	MYC	CYGB
23	GPX3	RAMP3	GSN	JUND	ABCA8
24	CXCL12	CD93	S100A6	KCNQ1OT1	CALD1
25	TIMP2	PLVAP	SCARA5	NAMPT	TMEM176B

26	FTL	HLA-DPA1	TIMP2	VEGFA	CRYBG3
27	CST3	IFI27	SFRP1	JUNB	STMN2
28	OGN	IGFBP7	GPX3	UAP1	LAMB1
29	PI16	SOCS3	C1R	MEDAG	IGFBP7
30	MFAP4	ZNF385D	LTBP4	ZFP36	CHL1

Cell cluster	Endothelial_ choriocapillary	Fibroblast_PTGDS_ APOE	Fibroblast_ DPT	SMC_ACTA2	Fibroblast_CXCL14_ F3_PDGFRA
1	FABP4	PTGDS	C7	TAGLN	CXCL141
2	PLVAP	APOE	DPT	MYH11	F3
3	FABP5	MGP	KCNN3	ACTA2	POSTN1
4	CD320	SFRP2	THBS4	MYL9	PDGFRA2
5	FLT1	GREM1	SCN7A	TPM2	PLAT
6	PECAM1	CFD	MGP	C11orf96	TRPA1
7	CD36	FTL	PDGFRA	MT1M	SOX6
8	CLDN5	CXCL12	RAMP1	ADIRF	NRG1
9	CD74	SERPINF1	LY6H	MT1X	EDNRB
10	APOLD1	IGLC2	TNC	MUSTN1	CALD12
11	TM4SF1	C3	P2RY1	DSTN	GADD45G
12	CAVIN2	RARRES2	GUCY1A1	MT2A	DMKN
13	CAV1	FTH1	FNDC1	FLNA	FENDRR
14	SPRY1	ADH1B	CCL11	CRIP1	ALKAL2
15	SPARCL1	LUM	LAMB1	MT1A	DDHD1
16	RGCC	DCN	CITED2	GADD45B	F2R
17	PIK3R3	CST3	MARCKS	MCAM	PTCH1
18	VWF	TIMP1	NFIA	TPM1	ENHO
19	EGFL7	FBLN1	COL6A3	LBH	BMP5
20	EPAS1	NUPR1	ADH1B	MT1E	PDGFD
21	CD93	IGLC3	OGN	SORBS2	FRZB1
22	RAMP2	CCDC80	MATN2	CSRP1	PCSK6
23	SLC9A3R2	RSPO3	PID1	MYLK	WNT5A

24	AQP1	IGKC	CXCL12	LMOD1	ADGRL31
25	RBP7	COL14A1	SULF1	ACTB	TMEM119
26	HLA-E	IGFBP4	SPRY2	CALD1	EMID11
27	IFI27	DEPP1	SMOC2	PLN	TGFBI1
28	THBD	SELENOP	ADAMTSL3	IGFBP7	KREMEN1
29	MGLL	C7	TSHZ2	SPARCL1	MT-CO12
30	SRGN	MMP2	PRKAR2B	LPP	CDH111
Cell cluster	Lymphoid_ Endothelial	Fibroblast_PLA2GA	Pericyte	Fibroblast_ECM	Endothelial_ arterial
1	CCL21	PLA2G2A	RGS5	COL1A1	IGFBP3
2	TFF3	CFD	STEAP4	COL3A1	CLDN5
3	MMRN1	SFRP2	IGFBP7	COL1A2	CAV1
4	LYVE1	TIMP1	COL4A1	SPARC	SRP14
5	AKAP12	MFAP5	ADAMTS4	COL6A3	PECAM1
6	CLDN5	DCN	ACTA2	COL6A1	TM4SF1
7	CAVIN2	C3	COL4A2	COL5A1	DEPP1
8	PPFIBP1	IGFBP6	CALD1	COL5A2	SRGN
9	TFPI	FTL	NOTCH3	COL6A2	IFI27
10	NTS	SERPINF1	RGS16	BGN	RAMP2
11	PROX1	CST3	EPS8	CTHRC1	KCTD12
12	PKHD1L1	PLAC9	NR2F2	MMP2	EPAS1
13	ATP5F1E	S100A4	SYNPO2	THY1	EDN1
14	KLF6	RARRES1	MYL9	LUM	KLF2
15	GNG11	RARRES2	NDUFA4L2	FSTL1	SLC9A3R2
16	CD9	PCOLCE	TAGLN	FBN1	ICAM2
17	NRP2	SFRP1	CD36	RCN3	SULF1
18	PTPRE	CD63	MYO1B	THBS2	PODXL
19	DSP	S100A10	ITGA1	MRC2	HLA-E
20	ECSCR	C1R	PDGFRB	SPON2	PTPRB
21	RELN	ADH1B	MAP1B	ELN	ID1

22	EGFL7	S100A11	IGFBP2	MMP23B	EGFL7
23	LAPTM5	MFAP4	MCAM	MXRA5	CLEC14A
24	ARL4A	SLPI	GJA4	VCAN	RNASE1
25	FLT4	FTH1	ADGRF5	SERPINH1	CAVIN2
26	HLA-E	RSPO3	AVPR1A	CPXM1	EFNB2
27	RHOJ	SEMA3C	CPE	CCDC80	CRIP2
28	PPP1R2	MGST1	COX4I2	COL12A1	CD93
29	TBX1	C16orf89	HES4	MEG3	FAM107A
30	RAB11FIP 1	IGSF10	GUCY1A2	COL16A1	STOM

Cell cluster	SMC_HHIP	SMC_DES_ ACTG2	Fibroblast_reticular_ cells	Fibroblast_MMP_W NT5A
1	HHIP	DES	CCL19	MMP1
2	MYH11	ACTG2	CCL21	MMP3
3	TAGLN	MYH11	RBP5	CXCL6
4	ACTA2	TAGLN	PTGDS	CHI3L1
5	MYLK	TPM2	APOE	CXCL5
6	TPM1	TPM1	TNFSF13B	TGFBI
7	CXCL14	MYL9	ADAMDEC1	COL7A1
8	TPM2	MYLK	C7	IL7R
9	LPP	FLNA	TMEM176B	WNT5A
10	NPNT	CNN1	CTSS	TMEM158
11	ACTG2	SYNM	SELENOM	CHI3L2
12	FLNA	SYNPO2	TYMP	INHBA
13	MYL9	CSRP1	CYP7B1	CD82
14	MYL6	ACTA2	IFI27L2	CCN4
15	DES	DSTN	VCAM1	MME
16	SYNPO2	CALD1	SOD2	TMEM132A
17	CSRP1	MYL6	FTH1	CXCL1
18	PTCH1	ACTB	CXCL14	CA12
19	SOSTDC1	LPP	C3	ACSL4
20	LMOD1	CKB	RARRES2	CFB

21	PDLIM3	RGS5	GEM	FAP
22	PALLD	SMTN	BIRC3	FAM20C
23	MYOCD	PALLD	TMEM176A	RAB31
24	EDIL3	LMOD1	RRAD	LOXL2
25	CNN1	SVIL	FAU	CXCL8
26	DMD	FLNC	SYNPO2	TNC
27	MRVI1	PRUNE2	B2M	PLAU
28	APOC1	PPP1R12B	CXCL3	COL3A1
29	NEO1	CARMN	DEPP1	C15orf48
30	ADGRL3	SORBS1	RACK1	LMO4

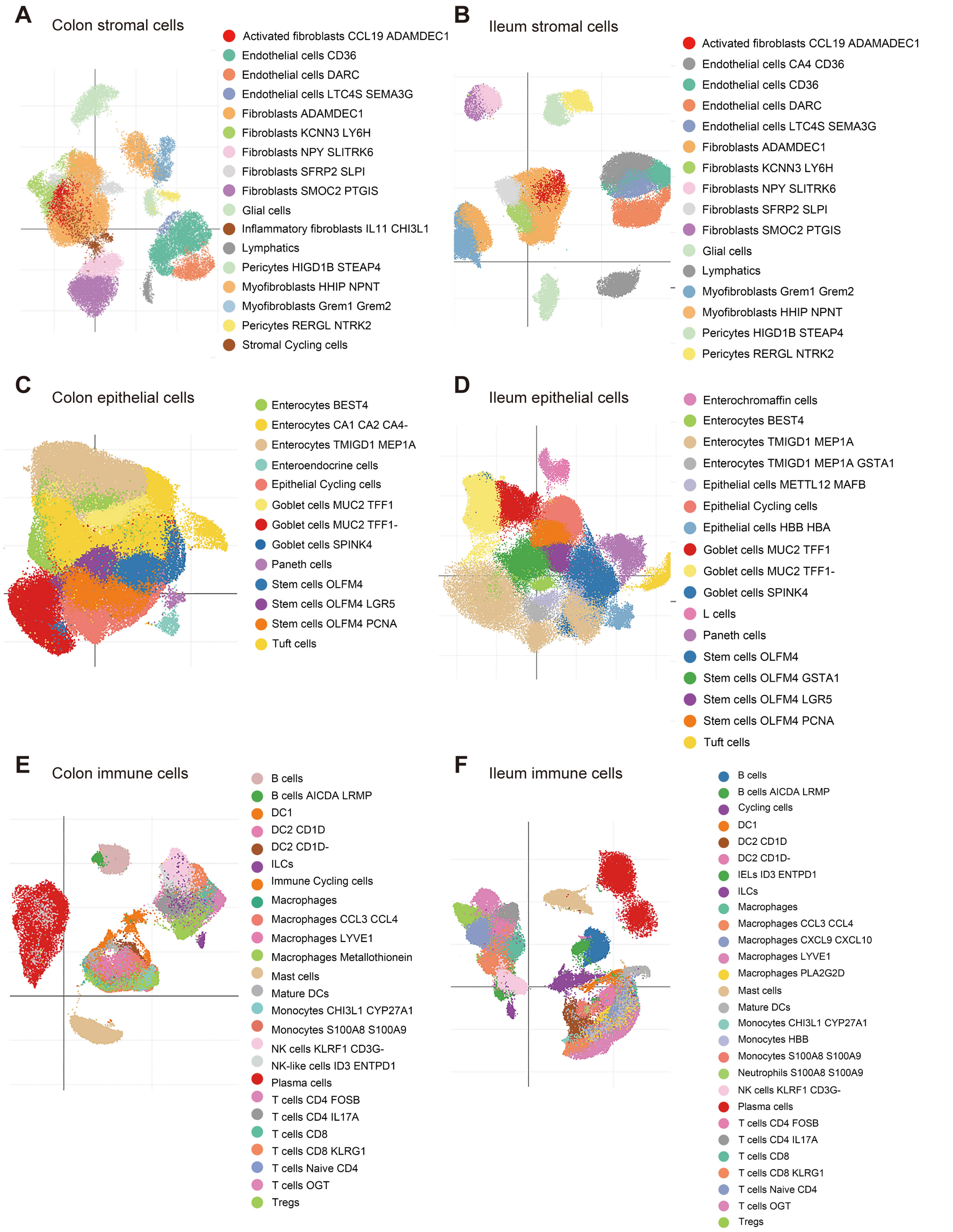

Supplemental Figure 1

Supplemental Figure 1. Reanalysis of scRNA-seq datasets of intestinal tissues from patients with Crohn's disease (CD)

(A-F) Uniform Manifold Approximation and Projection (UMAP) plots generated from a publicly available single-cell RNA sequencing (scRNA-seq) dataset of ileal and colonic

tissues obtained from patients with CD (Broad Single Cell Portal SCP1884). The plots display major cell clusters, including stromal **(A, B)**, epithelial **(C, D)**, and immune cell **(E, F)** populations.

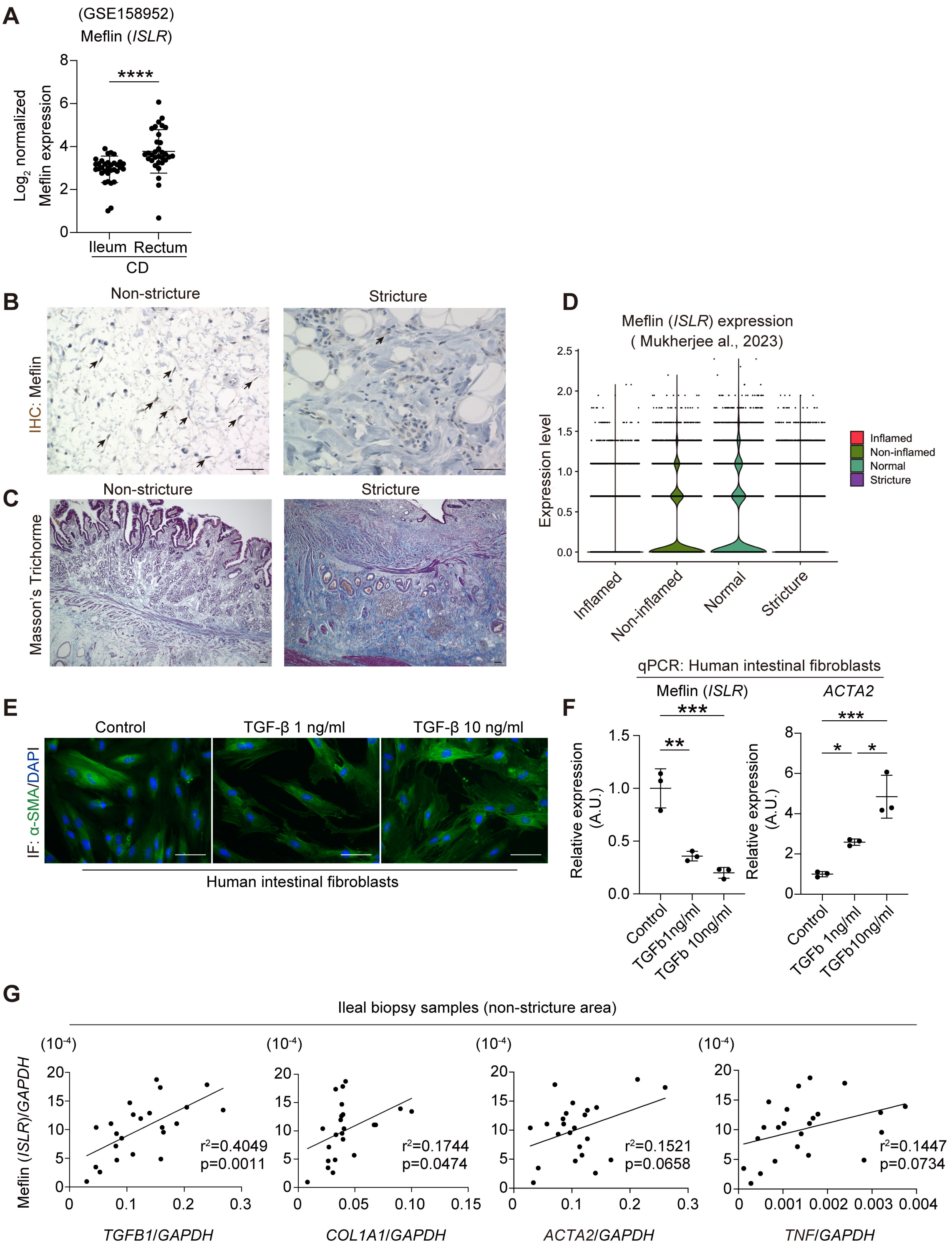

Supplemental Figure 2

Supplemental Figure 2. Downregulation of Meflin expression in fibroblasts in the intestinal stricture regions of patients with CD

(A) Normalized Meflin (*ISLR*) mRNA expression in biopsy samples obtained from the

ileum (n = 33) and rectum (n = 32) of patients with CD based on the reanalysis of a publicly available transcriptomic dataset (GSE158952).

(B) Representative images of Meflin immunohistochemistry (IHC) in tissue sections prepared from the non-stricture and stricture areas of the small intestine surgically resected from patients with CD, demonstrating preferential expression of Meflin in fibroblasts within the non-stricture areas. Arrows denote Meflin^{+} cells.

(C) Tissue sections prepared from the non-stricture and stricture areas are examined using Masson's trichrome staining, showing denser collagen deposition in the submucosal layer of the stricture area than in the non-stricture area.

(D) Violin plots show distinct Meflin (*ISLR*) expression in the intestines of patients with CD at different stages of intestinal fibrosis.

(E) Intestinal fibroblasts isolated from patients with CD were cultured either in the absence (control) or presence of TGF-β at 1 ng/mL (middle) or 10 ng/mL (right) for 24 hours, followed by immunofluorescent staining for α-SMA (green) and DAPI (blue). Representative images are shown. Scale bars, 100 µm.

(F) qPCR analysis for the expression of the indicated genes (*ISLR, ACTA2*) in human intestinal fibroblasts cultured by the indicated conditions (n = 3 per group).

(G) Pearson's correlation analysis of Meflin expression with *TGFB1, COL1A1, ACTA2*, and *TNF* expressions in ileal biopsy samples taken from the non-stricture regions of patients with CD (n = 24).

(A, F, G) Each dot represents an individual sample. Scale bars 40µm unless indicated. $^{*}p < .05$; ** $P < 0.01$, *** $p < .001$; **** $p < .0001$. For **(A),** two-tailed Student's *t* tests; for **(F)**, One-way ANOVA; for **(G)**, Pearson's correlation test.

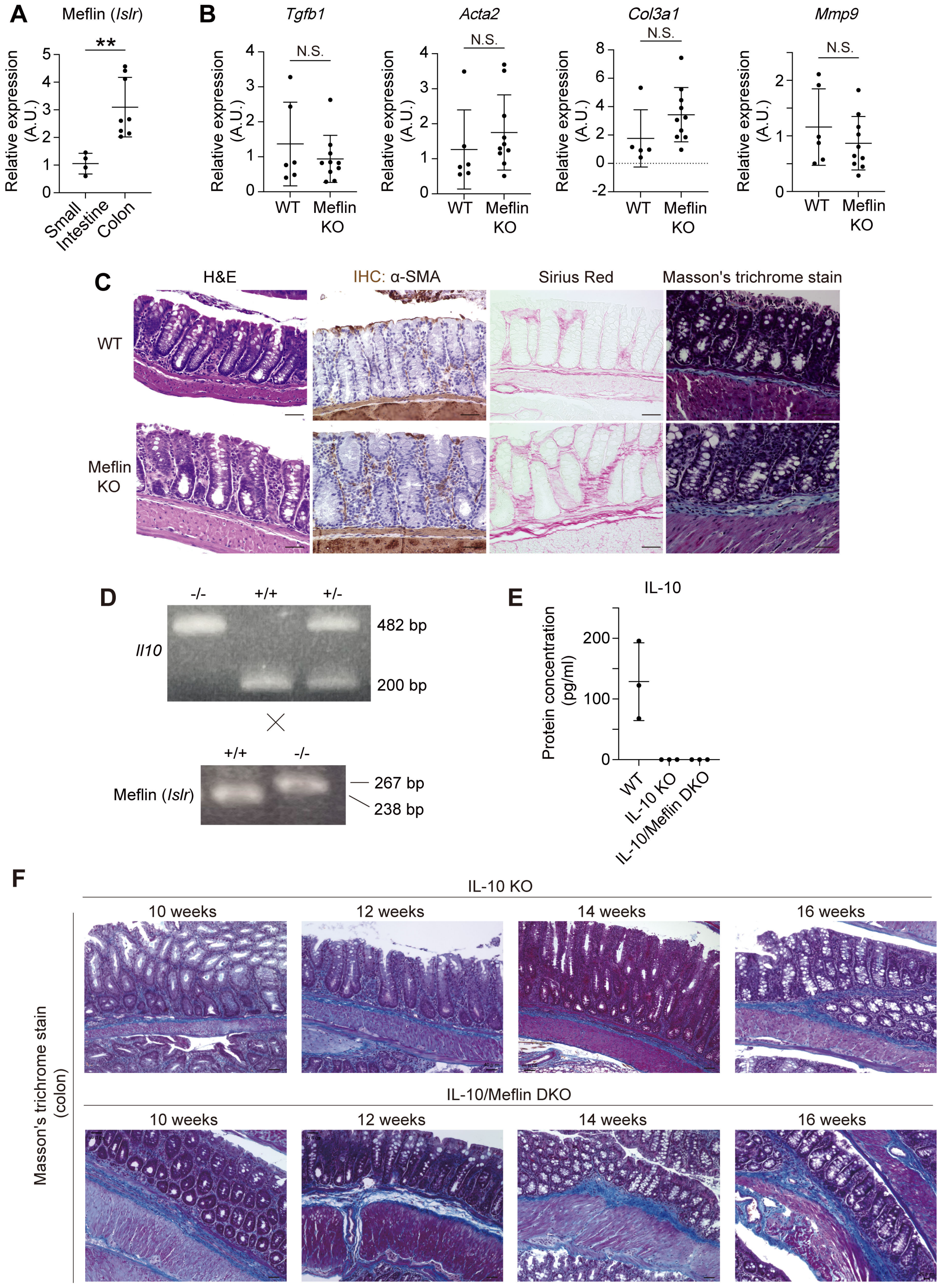

Supplemental Figure 3

Supplemental Figure 3. Characterization of Meflin-knockout (KO) and IL-10-KO mice used in the study

(**A**) Total RNAs were extracted from the colon (n = 8) and small intestine (n = 4) of

adult wild-type (WT) mice and examined by qPCR analysis for Meflin (*Islr*) mRNA expression.

(B) qPCR analysis for the expression of the fibrosis-related genes (*TGFb1*, *Acta2, Col3a1, and Mmp9*) in colonic tissues obtained from WT (n = 6) and Meflin-KO mice (n = 10).

(C) Representative images of H&E staining, IHC for a-SMA, Sirius Red staining, and Masson's trichrome staining on colon sections obtained from WT and Meflin-KO littermate mice of the same gender and age.

(D) Representative PCR genotyping data showing a deficiency of *Il10* (upper panel) and Meflin (*Islr*, lower panel) genes. The IL-10-mice were crossed with Meflin-KO mice to generate double-knockout (DKO) mice.

(E) Measurement of IL-10 protein levels in homogenates prepared from the colons of WT, IL-10-KO, and IL-10/Meflin DKO mice using ELISA (n = 3 per group).

(F) Representative Masson's trichrome-stained images of colon tissue sections from IL-10 (upper panels) and IL-10/Meflin-DKO mice.

(A, B, E) Each dot represents an individual sample. Scale bars, 40µm. ** P <0.01. Two-tailed Student's t tests.

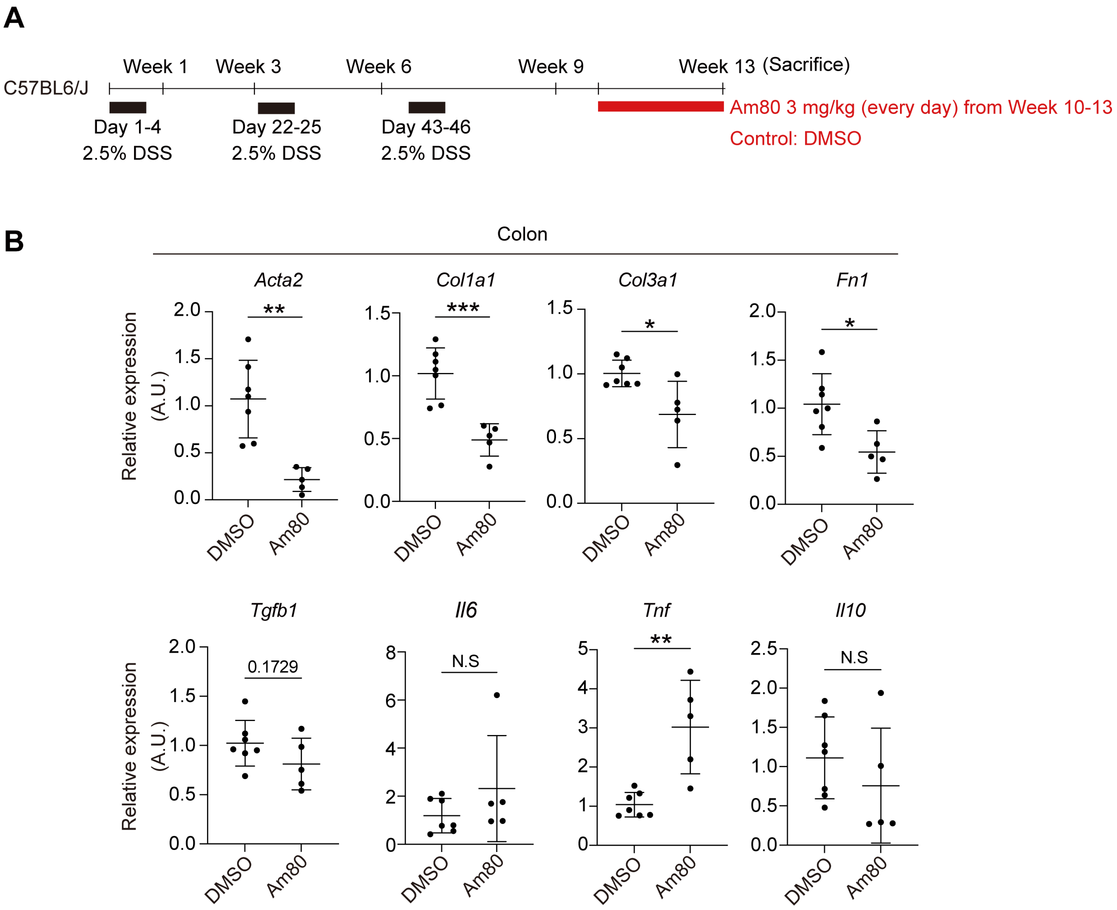

Supplemental Figure 4

Supplemental Figure 4. Am80 administration downregulated fibrosis-related gene expression in the DSS mouse model

(A) Schematic diagram showing the experimental setup.

(B) qPCR analysis for the expression of the indicated genes (*Acta2*, *Col1a1*, *Col3a1*, *Fn1*, *Tgfb1*, *Il6*, *Tnf*, *Il10*) in the colon tissues obtained from WT mice treated with DMSO or Am80 in the DSS mouse model.

(B) Each dot represents an individual sample. *P <0.05; ** p <.01; *** p <.001. Two-tailed Student's t tests.

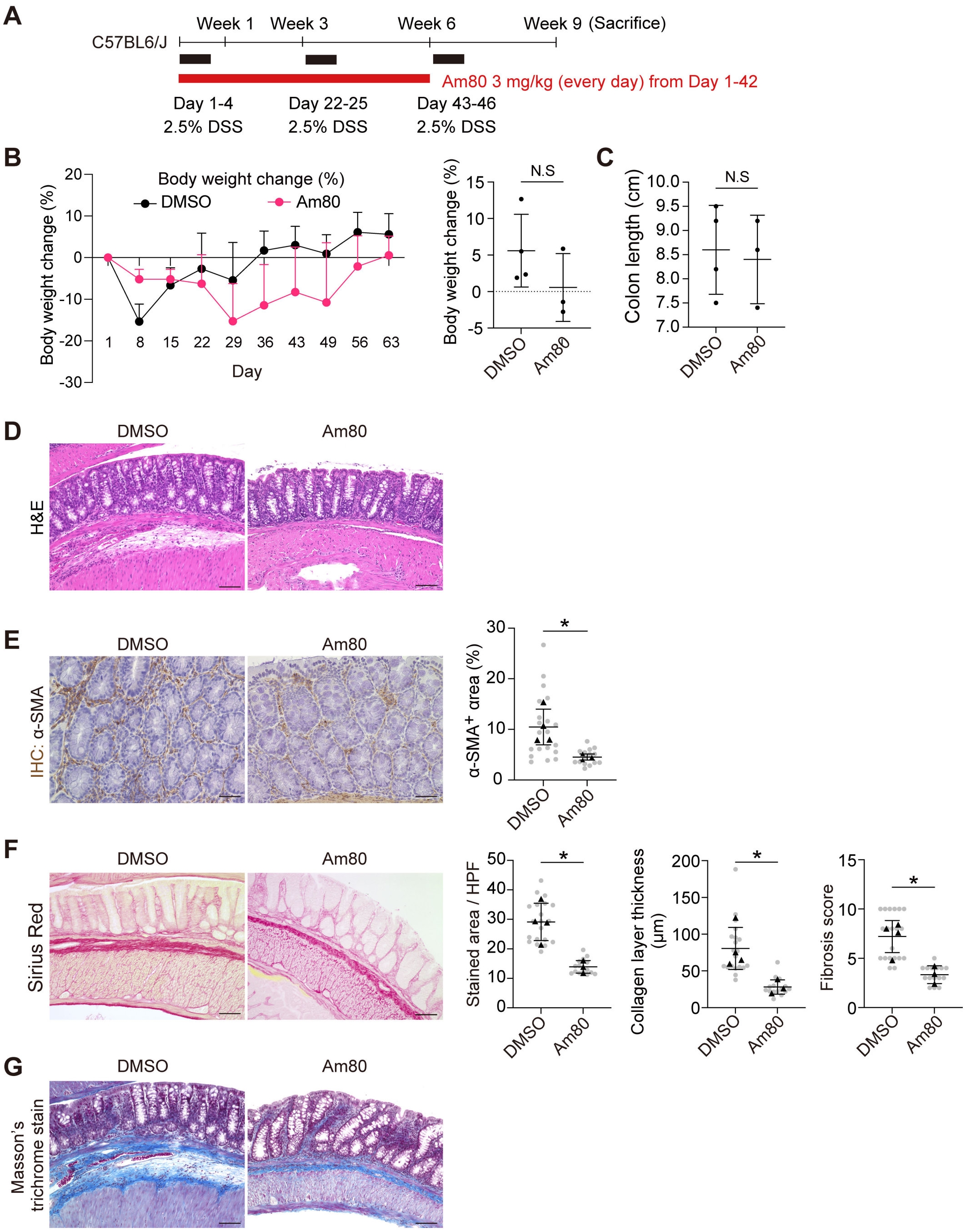

Supplemental Figure 5

Supplemental Figure 5. Am80 exerted a preventive effect against intestinal fibrosis in the DSS mouse model

(A) Schematic diagram showing the experimental setup.

(B, C) Body weight changes over time and colon length at Day 63 are measured,

followed by quantification.
(D-G) Colon tissue sections obtained from WT mice after Am80 administration were stained with H&E **(D)** and examined by IHC for a-SMA **(E)**, Sirius Red staining **(F)**, and Masson's trichrome staining **(G)**, followed by the quantification of collagen layer thickness, Sirius Red$^+$ areas, and fibrosis score (n = 4 and 3 for the control and Am80 groups, respectively).
(B, C) Each dot represents an individual sample. **(E, F)** Five HPFs per area were quantified for each mouse. Small gray dots indicate individual HPFs, and black triangles indicate mouse-level means used for statistical analysis. The DMSO-treated control samples in Supplemental Figure 5 are shared with those shown in Figure 5, and they are included here as a common control group for comparison with the additional treatment condition. Scale bars, 40 μm. Two-tailed Student's t tests. *P <0.05.

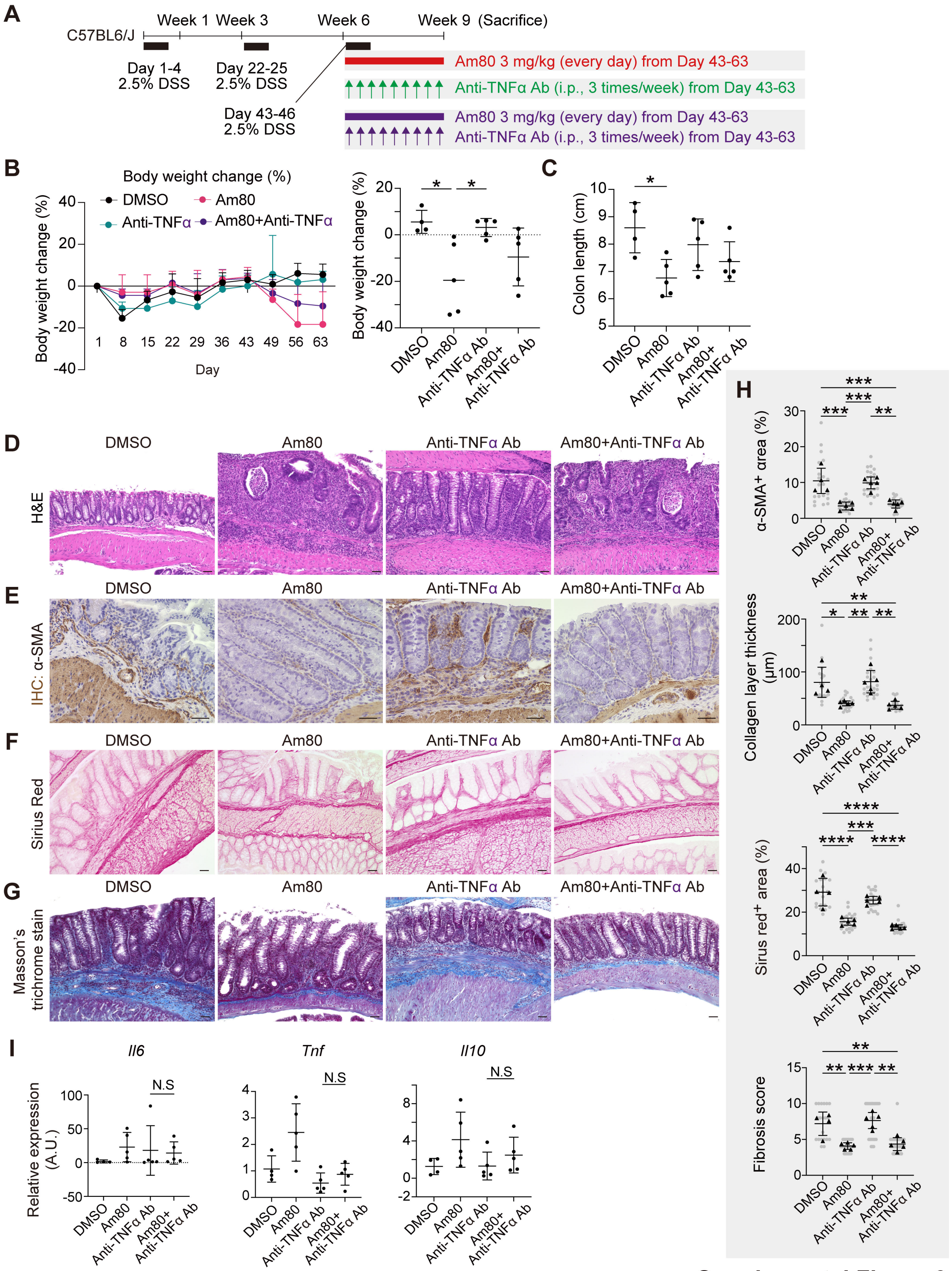

Supplemental Figure 6

Supplemental Figure 6. Effects of co-administration of Am80 and anti-TNFα antibodies on colitis and fibrosis in the DSS mouse model

(A) Schematic representation of the experimental setup. Am80 (3 mg/kg/day) or DMSO

is orally administered daily to WT mice treated with DSS, starting simultaneously from the third cycle of DSS administration (Days 43–63). Anti-TNF-α antibodies (10 mg/kg) are intraperitoneally (i.p.) administered three times per week alone or combined with Am80.

(B, C) Body weight changes over time and colon length at Day 63 are measured, followed by quantification.

(D-H) Colon tissue sections obtained from WT mice of the indicated treatment groups are stained with H&E **(D)**, Sirius Red **(F)**, and Masson's trichrome **(H)** and examined by IHC for a-SMA **(E),** followed by the quantifications of collagen layer thickness, Sirius Red^{+} areas, and fibrosis score **(H)** (n = 4 and 5 for the control and the treatment groups, respectively).

(I) qPCR analysis for the expression of the indicated genes (*Il6*, *Tnf*, *Il10*) in colonic tissues of WT mice following the indicated treatments.

(B right panel, C, I) Each dot represents an individual sample. **(H)** Five HPFs per area were quantified for each mouse. Small gray dots indicate individual HPFs, and black triangles indicate mouse-level means used for statistical analysis. The DMSO-treated and Am80-treated control samples in Supplemental Figure 6 are shared with those shown in Figure 5, and they are included here for comparison with the additional treatment condition. Scale bars, 40 μm. One-way ANOVA. **P*<0.05; ** *P*<0.01; *** *P*<0.001; **** *P*<0.0001.

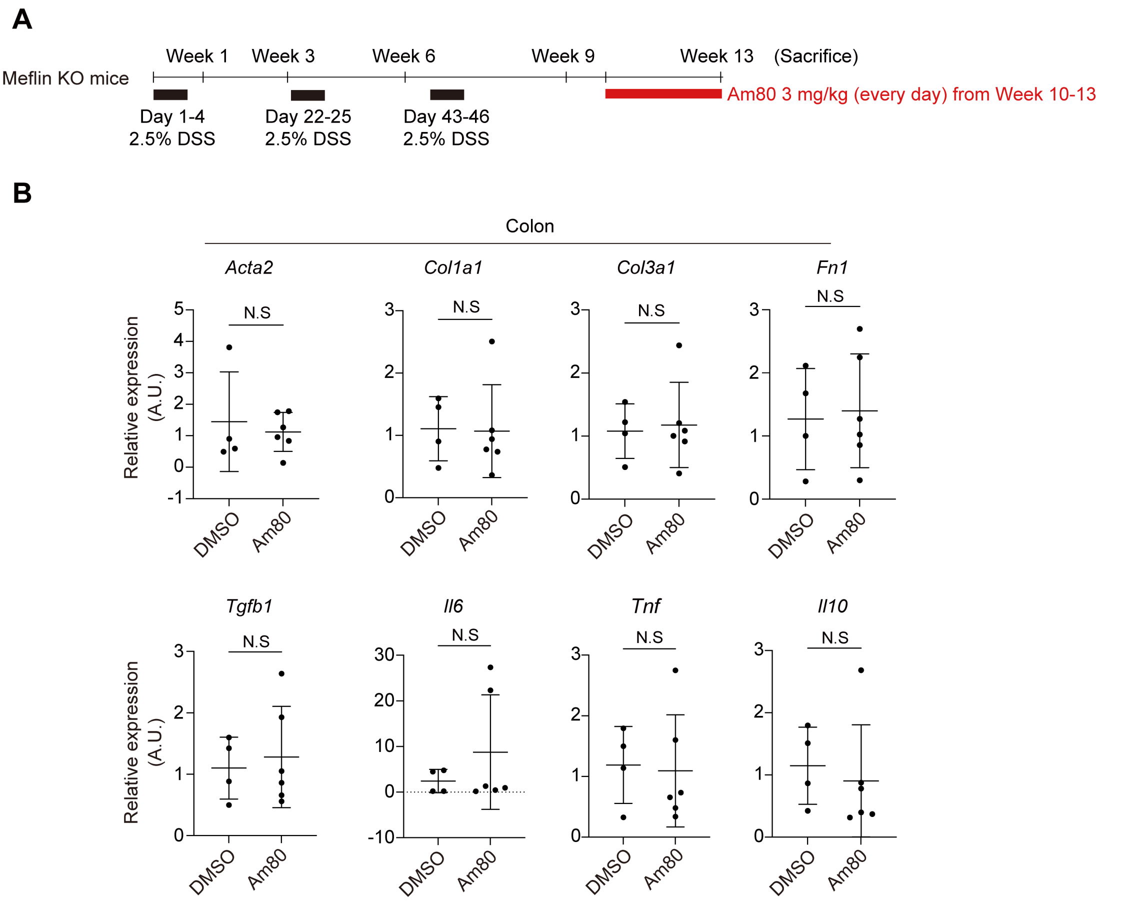

Supplemental Figure 7

Supplemental Figure 7. Meflin deficiency abolished the anti-fibrotic effect of Am80 treatment.

(A) Schematic representation of the experimental setup.

(B) qPCR analysis for the expression of the indicated genes (*Acta2*, *Col1a1*, *Col3a1*, *Fn1*, *Tgfb1*, *Il6*, *Tnf, and Il10*) in the colonic tissues of Meflin-KO mice after DMSO or Am80 administration (n = 4 and 6 for DMSO and Am80 groups, respectively).

(B) Each dot represents an individual sample. Two-tailed Student’s *t* tests.

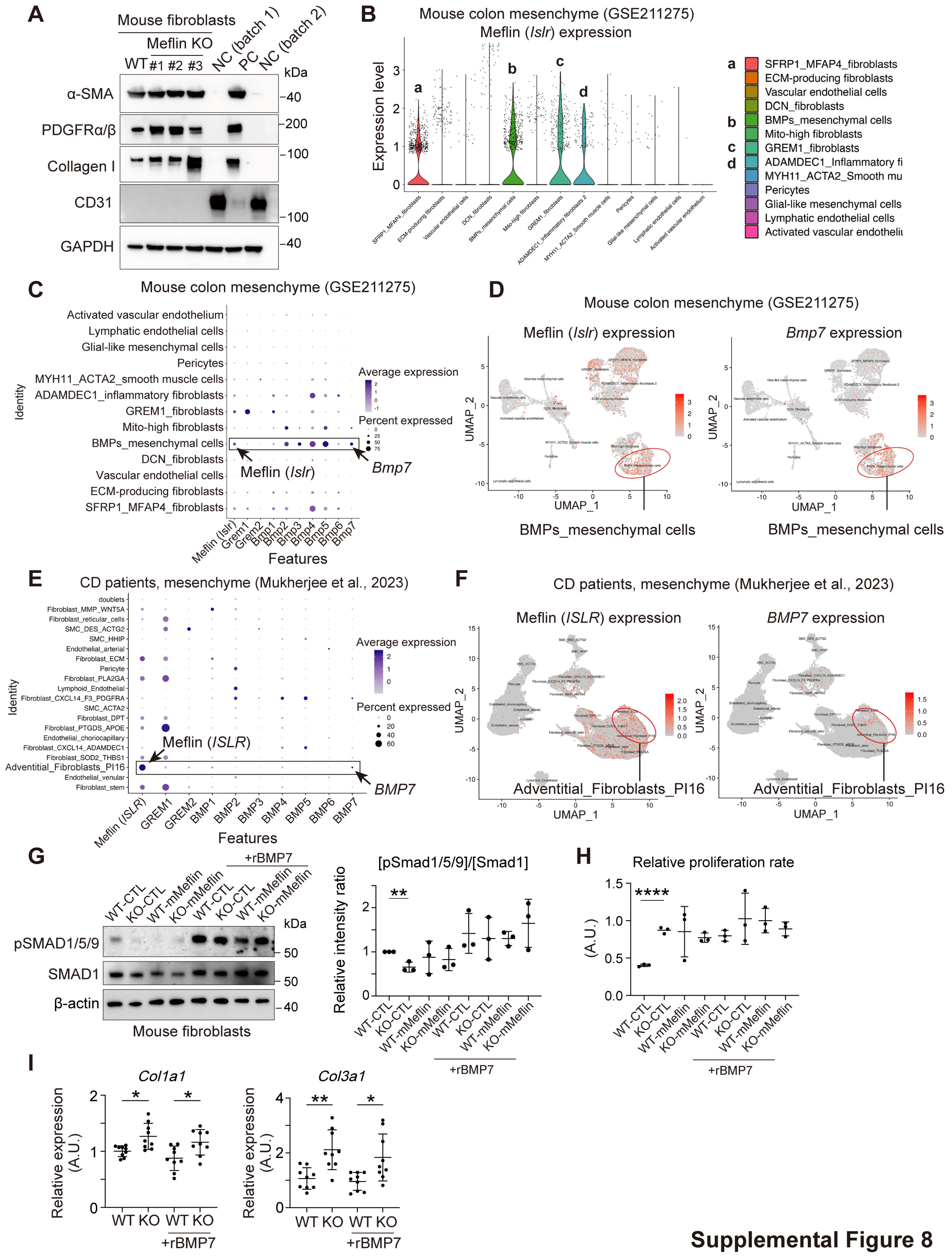

Supplemental Figure 8

Supplemental Figure 8. Limited involvement of BMP7 signaling in proliferation and collagen expression of Meflin-deficient fibroblasts

(A) Fibroblasts were isolated from the colons of adult WT and Meflin-KO mice and cultured on 6 cm^2 petri dishes to 90% confluency, followed by Western blot analysis.

Previously isolated mouse intestinal fibroblasts were used as the positive control (PC), while endothelial cells (batches 1 and 2) were used as the negative control (NC).

(B) Violin plots obtained by the reanalysis of the publicly available dataset of scRNA-seq of the mouse colon (GSE211275) show that Meflin (*Islr*) expression is enriched in the mesenchymal cell populations associated with BMP signaling.

(C-D) DotPlot **(C)** and UMAP plot **(D)** generated through the reanalysis of the scRNA-seq dataset (GSE211275) show that Meflin (*Islr*) and *Bmp7* are co-expressed in the indicated mesenchymal cell population in the mouse colon.

(E-F) DotPlot **(E)** and UMAP plot **(F)** generated through the reanalysis of the scRNA-seq dataset (Mukherjee et al., 2023) show that Meflin (*ISLR*) and *BMP7* are co-expressed in the indicated mesenchymal cell population in the ileum of patients with CD.

(G, H) Fibroblasts isolated from the colons of WT and Meflin-KO mice after 9 weeks of DSS administration were transduced with either control lentiviral expression vector (CTL) or that encoding mouse Meflin (mMeflin) and treated with or without recombinant BMP7 (rBMP7; 100 ng/ml) for 1 h, followed by Western blot analysis **(G)** and WST-1 assays **(H)** (n = 3 per group).

(I) Fibroblasts isolated from the colons of WT and Meflin-KO mice after 9 weeks of DSS administration were treated with or without recombinant BMP7 (rBMP7; 20 ng/ml) for 48 h, followed by qPCR analysis (n=9 per group).

(G, H, I) Each dot represents an individual sample. $^*P < 0.05$; $^{**} P < 0.01$; $^{****} P < 0.0001$. One-way ANOVA.

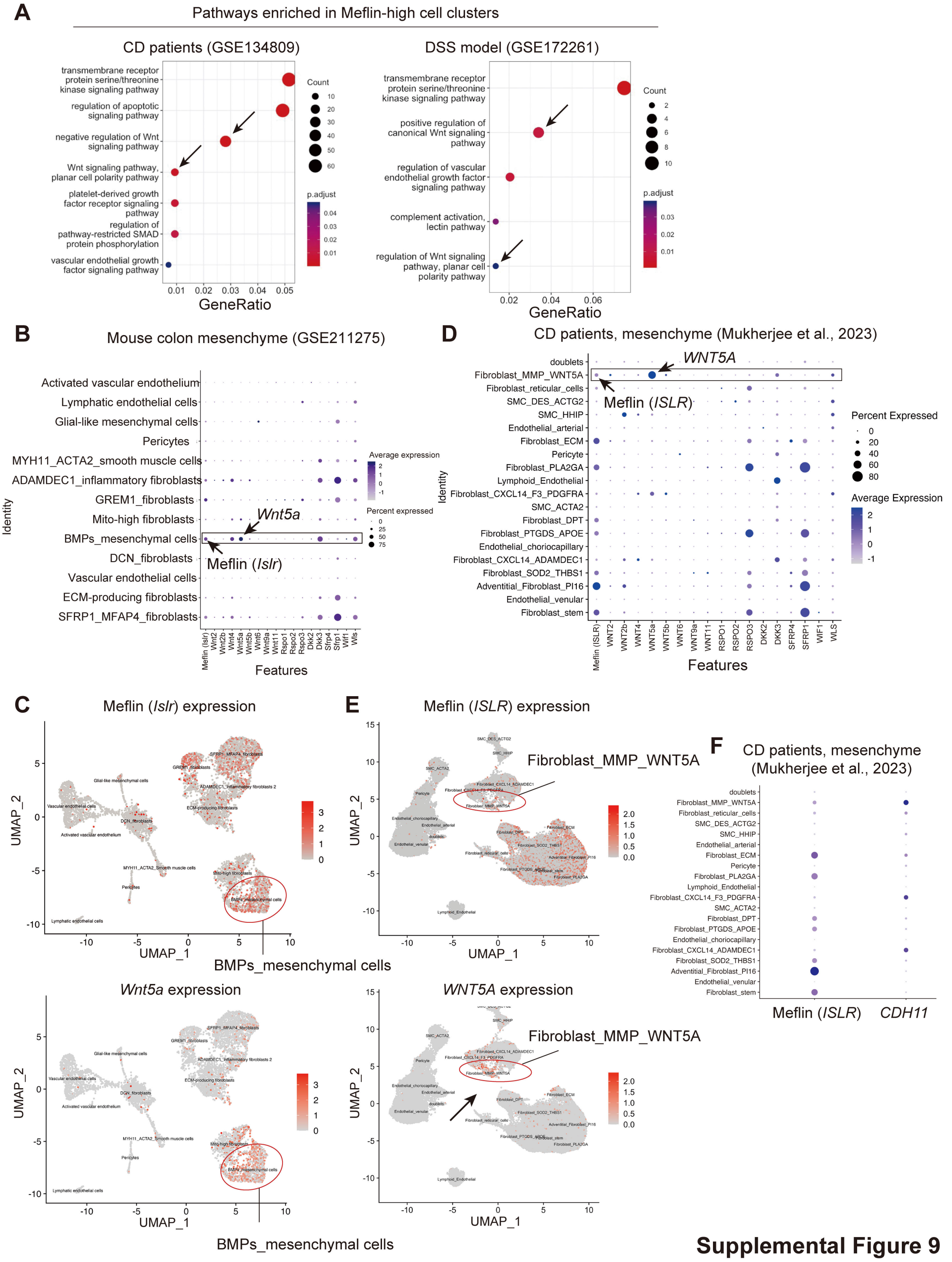

Supplemental Figure 9

Supplemental Figure 9. Enrichment of genes associated with the Wnt signaling pathway in Meflin-high fibroblasts derived from patients with CD and the DSS mouse model of colitis

(A) Gene ontology (GO) enrichment analysis is used to analyze the pathways enriched in Meflin-high fibroblasts in patients with CD (GSE134809) and chronic DSS mouse model (GSE172261). Arrows indicate signaling pathways associated with Wnt signaling.
(B-C) DotPlot **(B)** and UMAP plot **(C)** generated through the reanalysis of the scRNA-seq dataset (GSE211275) show that Meflin (*Islr*) and *Wnt5a* are co-expressed in the indicated mesenchymal cell population in the mouse colon.
(D-F) DotPlot **(D)** and UMAP plot **(E)** generated through the reanalysis of the scRNA-seq dataset (Mukherjee et al., 2023) show that Meflin (*ISLR*) and *WNT5A* are co-expressed in the indicated mesenchymal cell population in the ileum of patients with CD. **(F)** Meflin and *CDH11* are differentially expressed in the human mesenchymal cells, indicating that Meflin^{+} cells are distinct from CDH11^{+} cells, which have been reported to promote intestinal fibrosis (Mukherjee et al., 2023).

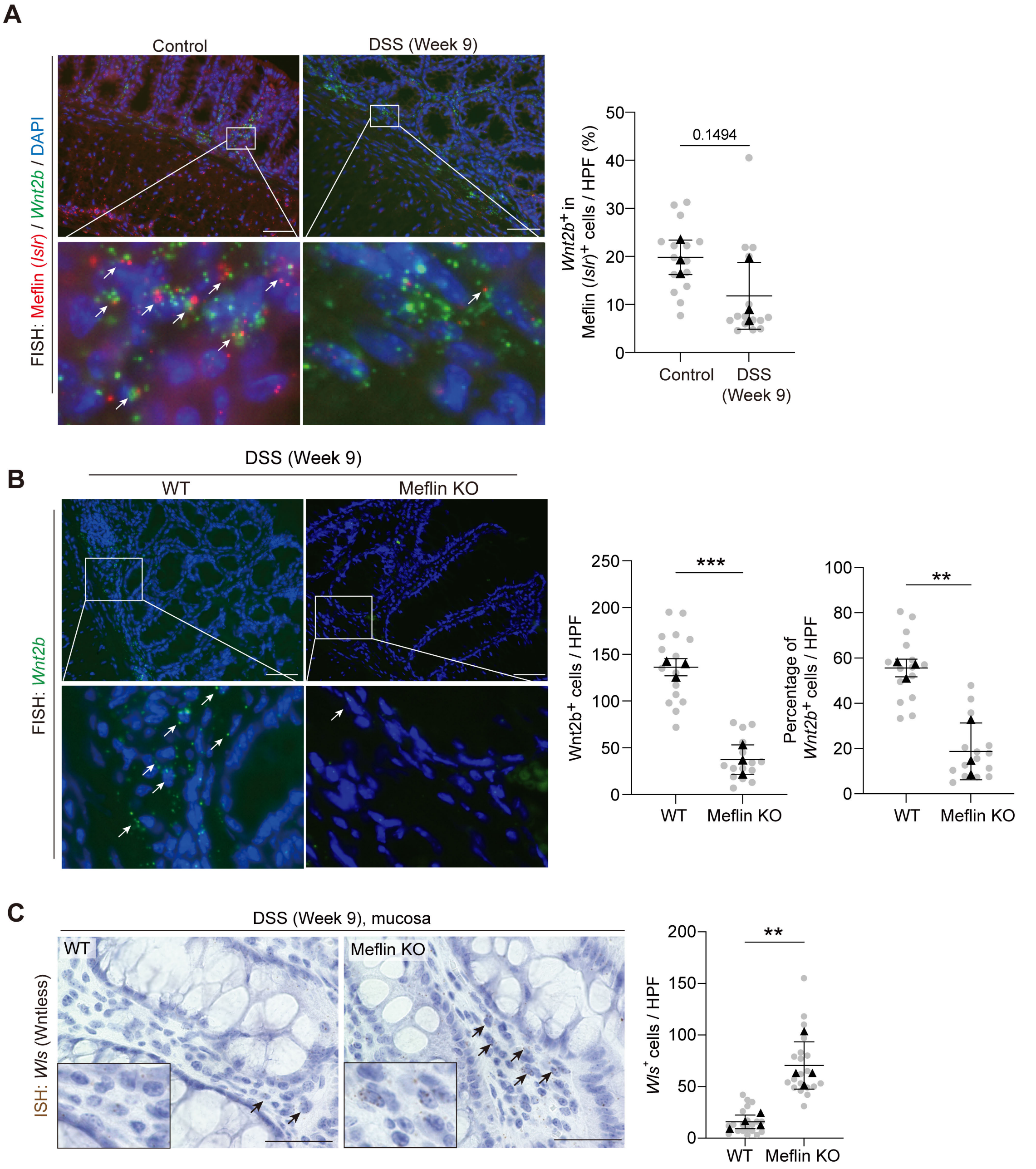

Supplemental Figure 10

Supplemental Figure 10. Downregulation of *Wnt2b* expression in the intestines of the DSS model and Meflin-KO mice

(A) Colon tissue sections prepared from the intestines of WT mice after 9 weeks of DSS administration were stained for Meflin (*Islr*, red) and *Wnt2b* (green) mRNA by fluorescence *in situ* hybridization (FISH) (left panel), followed by quantification of the

positivity of *Wnt2b* in Meflin+ fibroblasts (right panel). The boxed area is magnified in the lower panels. Arrows indicate *Wnt2b*+Meflin+ cells (n = 3 per group).

(B) Colon tissue sections prepared from the intestines of WT and Meflin-KO mice after 9 weeks of DSS administration were stained for *Wnt2b* by FISH. The boxed area is magnified in the lower panels. Arrows indicate *Wnt2b*-positive cells (n = 3 per group).

(C) Representative ISH images demonstrating upregulated *Wls* expression in the vicinity of intestinal crypt bases in Meflin-KO mice compared with WT mice (n = 4 per group).

(A–C) Five HPFs per area were quantified for each mouse. Small gray dots indicate individual HPFs, and black triangles indicate mouse-level means used for statistical analysis. Scale bars, 40 μm. Statistical significance was determined using two-tailed Student's t tests. ** $P < 0.01$; *** $P < 0.001$.

Supplemental Methods

Isolation and primary culture of intestinal fibroblasts

For primary human intestinal fibroblasts, freshly resected ileal tissue specimens obtained from the stenotic and non-stenotic regions of patients with CD were placed in ice-cold D-PBS supplemented with penicillin (100 U/mL) and streptomycin (100 μg/mL) and transported to a cell culture hood on ice. Intestinal fibroblasts were isolated from different regions, according to a previously published protocol. Briefly, samples were cut into small pieces and washed with ice-cold Hank's balanced salt solution (HBSS, Gibco, Life Technologies, CA) supplemented with penicillin and streptomycin by vigorously shaking the tube until the supernatants went clean. The cleaned samples were incubated with HBSS containing 5 mM ethylenediaminetetraacetic acid (Nacalai tesque, Japan) and 1 mM dithiothreitol (Roche, Germany) at 37°C for 20 min for removing the epithelium, followed by incubation in Dulbecco's modified Eagle medium (DMEM, Signa-Aldrich, USA) medium containing 2 mg/mL collagenase I (Wako) and 0.08 U/mL dispase II (Roche) for digestion at 37°C for 60 min in a shaking water bath. After centrifugation at 280 g for 10 min, cell pellets were resuspended in DMEM supplemented with antibiotics and 10% fetal bovine serum (Corning, CA) and placed in dishes, followed by culturing and passaging with the same medium. Primary mouse

intestinal fibroblasts were isolated according to the protocol described above, with a specific modification for Meflin-KO mice. Briefly, the digestion period with DMEM supplemented with 2 mg/mL collagenase I and 0.08 U/mL dispase II at 37°C was extended to 120 min to ensure complete tissue dissociation. Cells from passages 3 to 5 for both human and mouse were used in the experiments.

***Ex vivo* tissue culture**

Intestinal specimens were obtained from both stenotic and non-stenotic regions of the ileum surgically resected from seven patients with Crohn's disease (CD). Collected samples were placed in ice-cold RPMI1640 supplemented with penicillin (100 U/mL) and streptomycin (100 µg/mL) and transported to a cell culture hood immediately on ice. The specimens were cut into small pieces of approximately 1-2 mm^3 in size and washed at least three times in ice-cold RPMI1640 with penicillin and streptomycin until the supernatant became clear. After washing, the specimens were cultured with Am80 or an equal volume of dimethyl sulfoxide (DMSO). After 72 h of culturing, the supernatant was collected, centrifuged at 500 g for 10 min at 4°C, and stored for enzyme-linked immunosorbent assay (ELISA). For ELISA, protein concentrations were titrated using a BCA protein assay kit (T9300A, Takara, Shiga, Japan), and equal amounts of total

protein were subjected to ELISA. ELISA was performed according to the manufacturer's instructions. The ELISA kits used in this study were: Human IL-6 Immunoassay (D6050, R&D Systems, Minneapolis, MN, USA), Human TGF-β1 Immunoassay (DB100B R&D Systems), and Human IL-10 Immunoassay (D1000B, R&D Systems),

***In situ* hybridization (ISH)**

Formalin-fixed, paraffin-embedded (FFPE) human and mouse tissue samples were used for RNA *in situ* detection by the RNA scope technology. The HybEZ II Hybridization System (Advanced Cell Diagnostics, Newark, CA, USA) was used according to the manufacturer's instructions, along with the RNAscope 2.5 HD Reagent Kit – Brown (Advanced Cell Diagnostics) or RNAscope Multiplex Fluorescent Reagent Kit v2 (Advanced Cell Diagnostics), together with Opal fluorophores (NEL810001KT; Akoya Biosciences, Marlborough, MA, USA). Briefly, FFPE sections were baked at 60°C for 60 min in a dry oven. After deparaffinization and drying, slides were incubated with hydrogen peroxide for 10 min. The slides were then boiled in the target retrieval reagent for 30 min and incubated with protease plus solution at 40°C for 30 min, followed by incubation with probes for 4 h at 40°C and successively incubated with AMP regents.

Staining was visualized using the Liquid DAB+ Substrate Chromogen System (K3468, Dako, Santa Clara, CA, USA), followed by nuclear staining with hematoxylin. For multiplex fluorescence staining, the probes were mixed (C1:C2 = 50:1) before incubation, and staining was visualized using Opal 570 and Opal 690 (diluted 1:750 in TSA buffer), followed by nuclear staining with 4',6-diamidino-2-phenylindole (DAPI).The RNA scope probes used in this study were: Hs-ISLR (455481, Advanced Cell Diagnostics), Hs-ISLR-C2 (455481-C2, Advanced Cell Diagnostics), Hs-ACTA2-O1 (444771, Advanced Cell Diagnostics), Hs-PDGFRA-No-XMm (452051, Advanced Cell Diagnostics), Mm-Islr (450041, Advanced Cell Diagnostics), Mm-Islr-C2 (450041-C2, Advanced Cell Diagnostics), Mm-ACTA2 (319531, Advanced Cell Diagnostics), Mm-WNT5A (316791, Advanced Cell Diagnostics), Mm-Wnt2b (405031, Advanced Cell Diagnostics), Mm-Ror2 (430041, Advanced Cell Diagnostics), Mm-Wls (405011, Advanced Cell Diagnostics), Mm-Rspo3 (402011, Advanced Cell Diagnostics), and Mm-Axin2 (400331, Advanced Cell Diagnostics).

Immunohistochemistry (IHC)

All immunostaining analyses were performed on human and mouse FFPE tissue samples. After deparaffinization and rehydration, tissue sections were boiled at 98°C for

30 min in a pH 6 (S1699; Dako) or pH 9 (S2368, Dako) antigen retrieval solution, followed by cooling for 30 min. After washing with phosphate-buffered saline (PBS), sections were blocked with normal goat serum (MP7451, Vector Laboratories, Burlingame, CA, USA) for 30 min and then incubated with the indicated primary antibodies overnight at 4°C. The following day, endogenous peroxidase was blocked by incubating the sections in 0.3% hydrogen peroxide for 15 min, followed by incubation with horseradish peroxidase (HRP)-polymer-conjugated secondary antibodies (ImmPRESS reagent, HRP goat anti-rabbit IgG or goat anti-mouse IgG, Vector Laboratories). After washing with PBS, staining was visualized by incubating the sections with the Liquid DAB+ Substrate Chromogen System (K3468, Dako), followed by counterstaining with hematoxylin, and mounting and analysis under a microscope (BZ9000, Keyence, Osaka, Japan) (1).

For immunostaining mouse sections for α-smooth muscle actin (α-SMA), we used the mouse staining kit (414321, Histofine, Nichirei Bioscience, Tokyo, Japan) following the manufacturer's protocol that optimizes the procedure for staining mouse tissue sections with mouse primary antibodies. Briefly, after deparaffinization and rehydration, endogenous peroxidase was quenched with 3% hydrogen peroxide in absolute methanol for 15 min. Sections were blocked with blocking reagent A for 60 min at room

temperature. After washing, sections were incubated with primary antibodies at 4°C for 16 h. On the second day after washing, sections were incubated with blocking reagent B for 10 min, followed by incubation with Simple Stain Mouse MAX PO for 10 min. Finally, the staining was visualized using the DAB Substrate Chromogen System, followed by counterstaining with hematoxylin. Quantification data was calculated with 5 HPF/sample.

The primary antibodies used for IHC were mouse monoclonal anti-α-SMA antibody (dilution 1:1000, clone 1A4, Dako), rabbit polyclonal anti-Meflin antibodies (dilution 1:2500, HPA050811, Atlas antibody, Bromma, Sweden).

Immunofluorescent (IF) staining

4-well sterilized chamber slides (192-004, Watson, Tokyo, Japan) were used for IF staining of the cultured cells following the manufacturer's instructions. Briefly, chambers were coated with 50 μg/mL collagen type 1 (354236, Corning, Bedford, MA, USA) diluted in 0.02N acetic acid for 24 h at room temperature. Cultured fibroblasts were resuspended and placed in the collagen-coated chambers and cultured until 100% confluent, followed by the treatment with Am80 (1uM) or DMSO for 48 h cultured in the incubator. For IF staining, cells were fixed with 4% paraformaldehyde (PFA) for 15

min, followed by permeabilization with 0.1% Triton X-100 (Sigma-Aldrich, St. Louis, MO, USA) in PBS solution at room temperature for 5 min. The chambers were washed with Dulbecco's PBS (D-PBS) and blocked with 1% bovine serum albumin (Fraction V, 019-27051, Fujifilm Wako Chemicals, Osaka, Japan) diluted in D-PBS. After washing, cells were incubated with related primary antibodies for 1 h at room temperature, followed by second antibody incubation with Alexa Fluor 488-conjugated secondary antibodies (dilution 1:1000, 4412, Cell Signaling Technology) for 1 h. Finally, the chambers were mounted in 4′,6-diamidino-2-phenylindole Fluoromount-G (010020, Southern Biotech, Birmingham, AL, USA) and covered. Images were obtained using a fluorescence microscope (BZ-9000; Keyence).

The primary antibodies used for IF were mouse monoclonal anti-α-SMA antibody (dilution 1:1000, clone 1A4, Dako).

Masson’s trichrome staining

Masson’s trichrome staining was performed on human and mouse FFPE tissue sections using a staining kit (Muto Pure Chemicals, Tokyo, Japan), following the manufacturer’s instructions. Briefly, deparaffinized and rehydrated FFPE tissue sections were incubated with mordant solution (40061, Muto Pure Chemicals) for 30 min and washed with

running tap water for 3 min. Sections were stained with Weigert's iron hematoxylin (4034I, 4035-I; Muto Pure Chemicals) for 10 min. After rinsing in running tap water for 10 min, the sections were quickly treated with Second Mordant (81411, Muto Pure Chemicals) for 1 min and rinsed in tap water and 1% acetic acid as follows. The sections were then stained with 0.75% Orange G solution (40231; Muto Pure Chemicals) for 1 min, Masson's Stain Solution B (40251; Muto Pure Chemicals) for 25 min, 2.5% phosphotungstic Acid Solution (40182; Muto Pure Chemicals) for 20 min, and Aniline Blue Solution (40201; Muto Pure Chemicals) for 15 min. Sections were rinsed quickly in clear 1% acetic acid for a few seconds between each staining step. Finally, the sections were quickly washed with 1% acetic acid, dehydrated, cleared, mounted, and analyzed as described above.

Sirius Red staining

Sirius Red staining was performed on mouse FFPE tissue samples to visualize collagen deposition according to a standard protocol. Briefly, the tissue sections were deparaffinized, rehydrated, and treated with 2.5% sodium phosphomolybdate n-hydrate (194-0212, Fujifilm Wako Chemicals) for 30 s. After rinsing with tap water, the samples were stained with 1% Sirius Red (196-16201, Fujifilm Wako Chemicals) for 3 min and

quickly rinsed again with running tap water. The sections were dehydrated and mounted as described above. The measurement of collagen layer thickness, quantification, and calculation of Sirius Red$^+$ areas (5 HPF/sample) and fibrosis score (2) were determined based on Sirius Red staining.

Image acquisition and analysis

Images were obtained using a universal fluorescence microscope (BZ9000; Keyence, Osaka, Japan). For quantitative analysis of the stained positive area in IHC and Sirius Red staining, functions "Color Deconvolution" and "Threshold" were utilized. The measurement of the thickness of submucosa and collagen thickness was performed by using the function “Straight line" and "Measurement." For ISH analysis, positive cells were calculated using the "Analyze Particles" function. Images were analyzed using Image J/Fiji software.

RNA extraction and quantitative PCR

Freshly resected intestinal tissue samples from patients with CD were obtained as described above and preserved in RNAlater (R0901; Sigma-Aldrich, St. Louis, MO, USA). Total RNA was extracted using the standard acid guanidinium thiocyanate-

phenol-chloroform extraction method. To analyze alterations in gene expression, specifically in primary human intestinal fibroblasts, total RNA from cultured cells was extracted using the RNeasy Mini Kit (74104, Qiagen, Germany) according to the manufacturer's instructions. Accurate quantification of all extracted RNA samples was performed using a NanoDrop 1000 Spectrophotometer (Thermo Fisher Scientific, Waltham, MA, USA) before reverse transcription into cDNA using ReverTra Ace (Toyobo, Tokyo, Japan). Real-time quantitative PCR (qPCR) was performed using TaqMan Gene Expression Master Mix (Applied Biosystems, Foster City, CA, USA) in an Mx3000P thermal cycler (Agilent Technologies) or Aria MX real-time PCR system (Agilent Technologies). Cycling conditions were as follows: 50°C for 2 min, 95°C for 10 min, 45 cycles of 95°C for 15 s, and then 60°C for 1 min. Data were analyzed using the 2-ΔΔ comparative threshold cycle (CT) method and normalized to the Gapdh control. TaqMan probes and primers were purchased from Life Technologies (Carlsbad, CA, USA) and used according to the manufacturer's instructions. The primers used in this study were: Human ISLR (Hs01921558_s1), Human ACTA2 (Hs00426835_g1), Human TGFB (Hs00998133_m1), Human COL1A1 (Hs00164004_m1), Human IL10 (Hs00961622_m1), Human GADPH (Hs99999905_m1), Mouse Islr (Mm01700423_m1), Mouse Tnf (Mm00443258_m1), Mouse Tgfb1

Mm01178820_m1), Mouse Il6 (Mm00446190_m1), Mouse Col1a1 (Mm00801666_g1), Mouse Col3a1 (Mm00802300_m1), Mouse Col6a1 (Mm00487160_m1), Mouse Il10 (Mm01288386_m1), Mouse Acta2 (Mm00725412_s1), Mouse Ror2 (Mm00443470_m1), Mouse Fn1(Mm01256744_m1), Mouse Mmp9 (Mm00442991_m1), and Mouse Gapdh (Mm99999915_g1).

Western blot analysis (WB)

Cells were lysed in 2x Laemmli sample buffer (Bio-Rad, #1610737) with 5% 2-mercaptoethanol for 5 min at 95°C and separated by SDS-polyacrylamide gel electrophoresis. Proteins were transferred to nitrocellulose membranes, blocked in 5% milk in 0.1% tTBS (Nacalai Tesque), incubated with primary antibodies, and detected by horseradish peroxidase (HRP)-linked secondary antibodies (Cell Signaling Technology). The primary antibodies used for WB were: rabbit polyclonal anti-Meflin antibodies (dilution 1:1000, HPA050811, Atlas antibody, Bromma, Sweden), Wnt5a/b (C27E8) rabbit monoclonal antibody (dilution 1:1000, #2530, Cell Signaling Technology), anti-α-SMA antibody (dilution 1:2000, clone 1A4, Dako), COL1A1 Antibody (dilution 1:1000, B17N21, Selleckchem), PDGFR α/β Antibody (dilution 1:5000, C19P10, Selleckchem), CD31 (PECAM-1) antibody (dilution 1:1000, M18G16,

Selleckchem), phospho-SMAD1 (Ser463/465)/ SMAD5 (Ser463/465)/ SMAD9 (Ser465/467) (D5B10) rabbit monoclonal antibody (dilution 1:1000, #13820, Cell Signaling Technology), SMAD1 antibody (dilution 1:1000, #9743, Cell Signaling Technology), GAPDH (14C10) rabbit monoclonal antibody (dilution 1:1000, #2118, Cell Signaling Technology), beta-Actin Antibody (dilution 1:1000, #4967, Cell Signaling Technology).

Cell proliferation assay

For rBMP7 treatment, primary mouse intestinal fibroblasts were seeded and grown to 90% confluence. For acute stimulation, cells were serum-starved for 12 h and subsequently treated with rBMP7 (100 ng/mL; R&D Systems, #5666-BP) for 1 h before harvesting. Alternatively, for chronic treatment, fibroblasts were cultured with rBMP7 (20 ng/mL) for 48 h without prior starvation. Following treatment, total RNA and protein were extracted for Western blot and qPCR analysis as described below.

Database analysis

We utilized public transcriptomic data from the Gene Expression Omnibus (GEO) under accession numbers GSE165512 and GSE83687 to assess meflin (ISLR) transcript levels

in the intestinal tissues of healthy individuals (n = 80) and patients diagnosed with CD (n = 48). Biopsy samples were specifically obtained from colonic sites. Log2-transformed normalized counts were extracted via GEO2R and visualized using Prism 9 software (GraphPad, San Diego, CA, USA).

To visualize ISLR expression, we used the Single Cell Portal (https://singlecell.broadinstitute.org/single_cell, SCP1884) analyzing the colonic expression pattern and distribution of *ISLR*, *PDGFRA*, *PDPN*, and *ACTA2* in patients with CD using uniform manifold approximation and projection (UMAP) dimensionality reduction.

To explore the transcriptomic landscape of Meflin-expressing cells, we analyzed published scRNA-seq datasets, including a mouse mesenchymal cell database (GSE211275) and a human Crohn's disease (CD) patient database (Mukherjee et al., Gastroenterology, 2023). Briefly, we followed the standard Seurat workflow for data integration, normalization, and dimension reduction. To determine the optimal clustering parameters, silhouette scores were calculated to reflect the superior clustering performance, and the stability of these clusters was further confirmed using the Clustertree function. The final resolution provided with the highest silhouette score and a consistency of cluster branching in the cluster tree was selected. Following clustering,

the FindMarkers function was utilized to identify cluster-specific marker genes and define cell identities. The expression levels of *ISLR* (encoding Meflin) across all identified clusters were visualized using violin plots. Furthermore, to investigate the correlation between Meflin and its potential downstream effectors, the expression patterns and spatial distribution of co-expressed genes were represented using dot plots and UMAP plots, respectively.

To characterize the biological pathways associated with high Meflin expression, we performed Gene Ontology (GO) enrichment analysis on ISLR/Islr-high cell clusters identified from the human CD (3) (GSE134809) and chronic DSS (4) (GSE172261) datasets (5). Cells with an expression level of ISLR/Islr > 3 were stratified as the 'Meflin-high' cluster. Differentially expressed genes (DEGs) between Meflin-high and Meflin-low clusters were identified, and functional enrichment was conducted using the enrichGO function in the clusterProfiler R package (v4.6.2) (6-8). To refine the results, redundant GO terms were merged and filtered using the simplify function. All bioinformatic analyses were performed using the Seurat R package (v4.3.0) within the RStudio (2022.12.0+353 version) environment.

Chronic dextran sulfate sodium-induced mouse model of colitis

Chronic intestinal inflammation and fibrosis were induced by three cycles of 2.5% dextran sulfate sodium (DSS, MP Biomedicals, Ohio, USA) in drinking water for five consecutive days, followed by 16 days of normal sterile drinking water. The mice were euthanized at the designated time points. Changes in body weight and colon length were evaluated in all the *in vivo* experiments. Histological evaluation and pathologic scoring were performed blinded based on standard hematoxylin and eosin (H&E) staining (9). All the experiments were performed and reported in accordance with ARRIVE guidelines.

Supplemental references

1. Sakai A, et al. Increased Meflin expression in cancer-associated fibroblasts restrains tumor cell proliferation and shapes vessel-rich stroma in triple-negative breast cancer. *Am J Pathol* .2026:S0002-9440(26)00031-3.

2. Theiss AL, et al. Growth hormone reduces the severity of fibrosis associated with chronic intestinal inflammation. *Gastroenterology.* 2005;129(1):204-19.

3. Martin JC, et al. Single-cell analysis of Crohn's disease lesions identifies a pathogenic cellular module associated with resistance to anti-TNF therapy. *Cell.* 2019; 178:1493–1508.e20.

4. Jasso GJ, et al. Colon stroma mediates an inflammation-driven fibroblastic response controlling matrix remodeling and healing. *PLOS Biol.* 2022;20: e3001532.

5. Hao Y, et al. Integrated analysis of multimodal single-cell data. *Cell.* 2021; 184:3573–3587.e29.

6. Wu T, et al. clusterProfiler 4.0: A universal enrichment tool for interpreting omics data. *Innovation (Camb).* 2021; 2:100141.

7. Xu S, et al. Using clusterProfiler to characterize multiomics data. *Nat Protoc.* 2024; 19:3292–320.

8. Yu G, et al. clusterProfiler: An R package for comparing biological themes among gene clusters. *Omics.* 2012; 16:284–287.

9. Wirtz S, et al. Chemically induced mouse models of acute and chronic intestinal inflammation. *Nat Protoc*. 2017;12(7):1295–1313.
