# Supplementary material for: Meflin confers antifibrotic properties to intestinal fibroblasts in inflammatory bowel disease
Source: J Clin Invest. 2026 May 19;136(13):e192804. doi: 10.1172/JCI192804 (PMC13318116; doi:10.1172/JCI192804)

Figure 8C WNT5A/B

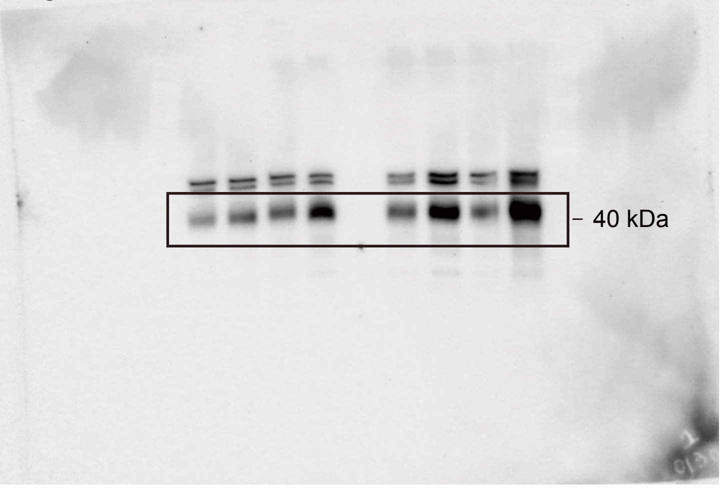

Figure 8C β-actin

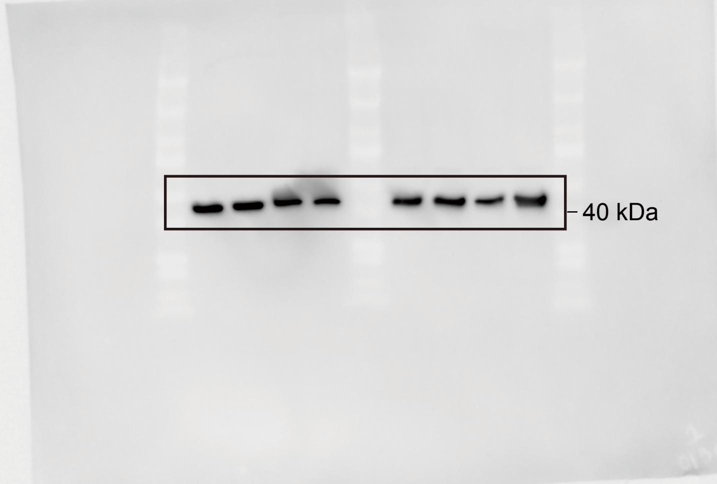

Figure 8D Meflin

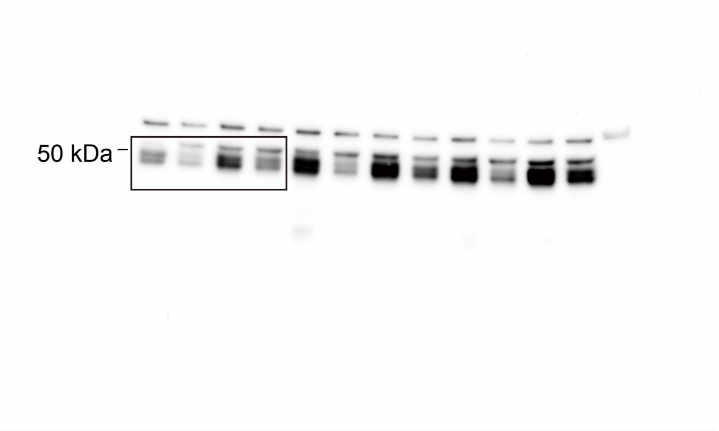

Figure 8D Wnt5a/b

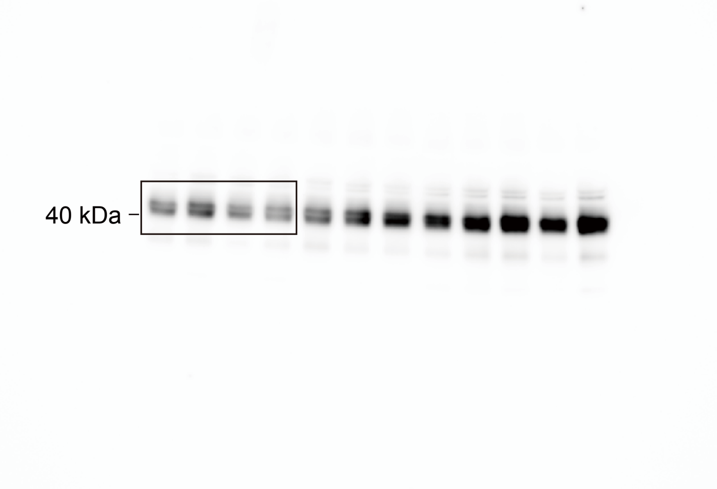

Figure 8D Gapdh

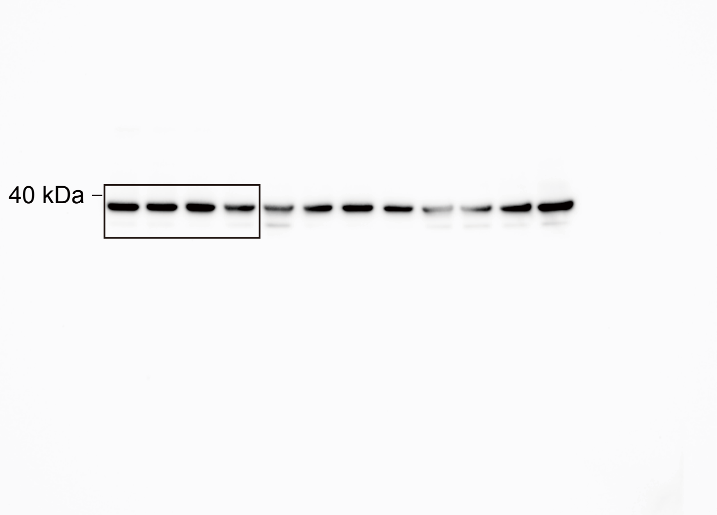

Supplemental Figure 8A a-SMA

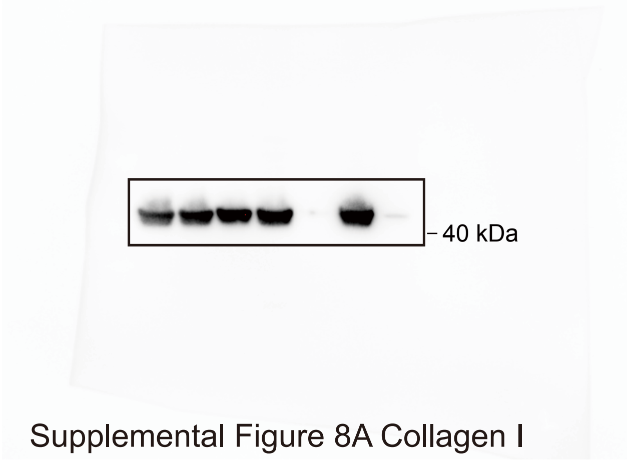

Supplemental Figure 8A PDGFRa

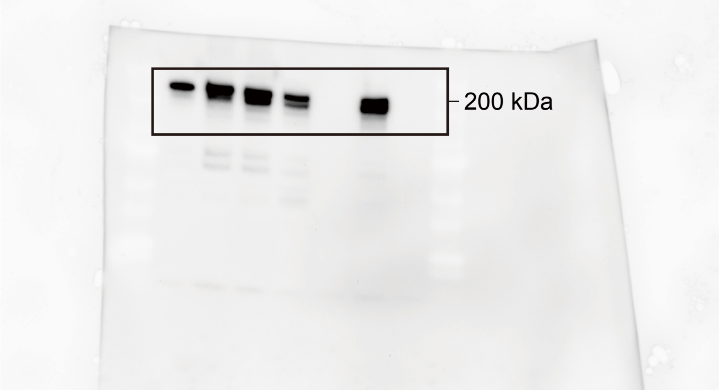

Supplemental Figure 8A Collagen I
(Cleaved fragements from COL1A1)

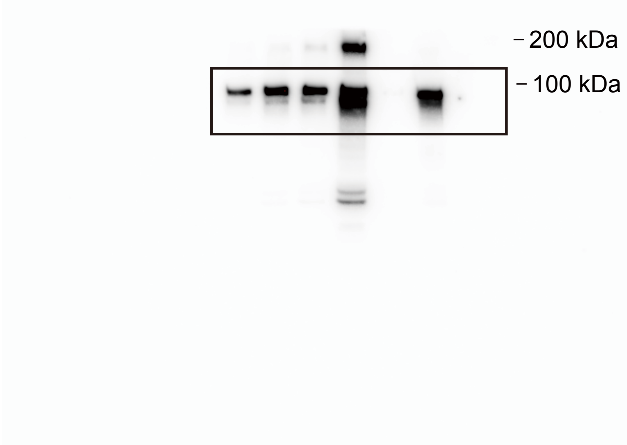

Supplemental Figure 8A CD31

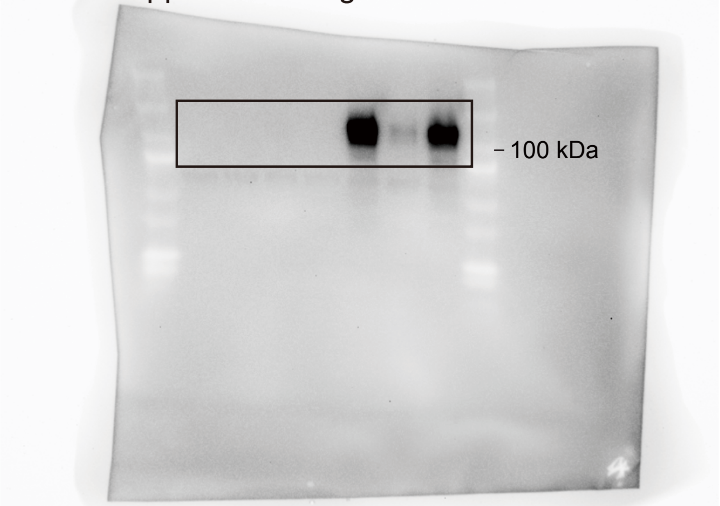

Supplemental Figure 8G pSmad1/5/9

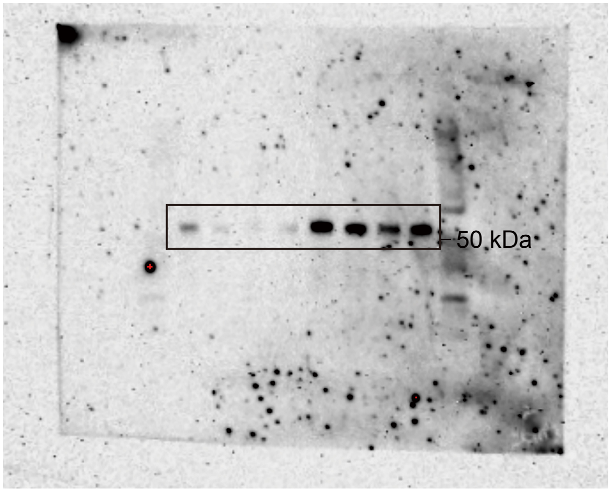

Supplemental Figure 8A GAPDH

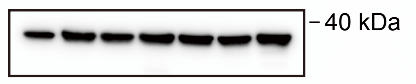

Supplemental Figure 8G Smad1/5/9

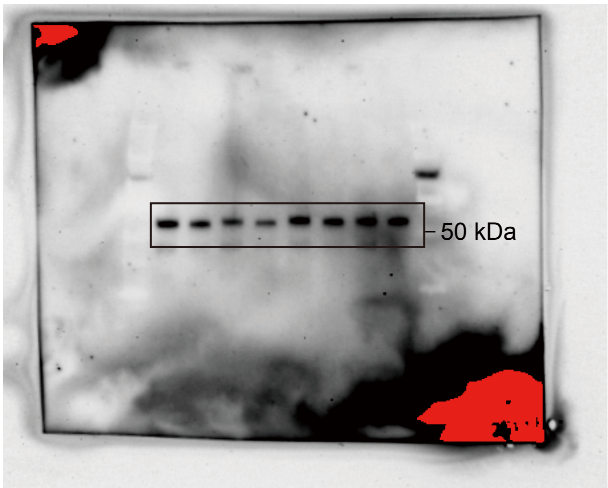

Supplemental Figure 8G β-actin

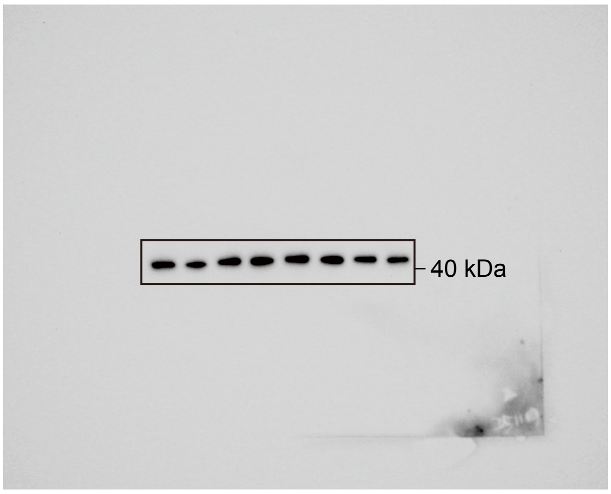

Supplement: Unedited blot and gel images [file jci-136-192804-s218.pdf]
